# Clinical-grade human dental pulp stem cells improve adult hippocampal neural regeneration and cognitive deficits in Alzheimer's disease

**DOI:** 10.7150/thno.102315

**Published:** 2025-01-01

**Authors:** Wei Xiong, Wenting She, Ye Liu, Heng Zhou, Xinxin Wang, Fang Li, Ruohan Li, Junnan Wang, Dongdong Qin, Shuili Jing, Xingxiang Duan, Cailei Jiang, Chun Xu, Yan He, Zhihao Wang, Qingsong Ye

**Affiliations:** 1 Center of Regenerative Medicine, Department of Stomatology, Renmin Hospital of Wuhan University, Wuhan University, Wuhan, China.; 2 Institute of Regenerative and Translational Medicine, Tianyou Hospital, Wuhan University of Science and Technology, Wuhan, China.; 3 Center for Neurodegenerative Disease Research, and Department of Neurology, Renmin Hospital of Wuhan University, Wuhan, China.; 4 Sydney School of Dentistry, The University of Sydney, Sydney, NSW, Australia.; 5 Department of Orthopedics, The Second Affiliated Hospital of Nanchang University, Nanchang, China.

**Keywords:** Alzheimer's disease, Stem cell-based therapies, Human dental pulp stem cells, Neural regeneration, Wnt/β-catenin pathway

## Abstract

**Background:** Disrupted hippocampal functions and progressive neuronal loss represent significant challenges in the treatment of Alzheimer's disease (AD). How to achieve the improvement of pathological progression and effective neural regeneration to ameliorate the intracerebral dysfunctional environment and cognitive impairment is the goal of the current AD therapy.

**Methods:** We examined the therapeutic potential of clinical-grade human derived dental pulp stem cells (hDPSCs) in cognitive function and neuropathology in AD. Specifically, we investigated the effect of neural crest-specific derived hDPSCs on endogenous neural regeneration and long-term efficacy following a single transplantation in the triple-transgenic mouse model (3xTg-AD).

**Results:** Our research demonstrated that a single administration of clinical-grade hDPSCs yielded dramatic short-term therapeutic benefits (5 weeks) and sustained partial efficacy (6 months) with respect to improving cognitive impairment and delaying typical pathological progression in 3xTg-AD mice. Intriguingly, exogenous hDPSCs were robustly self-differentiated into newborn functional neurons in the hippocampus of 3xTg-AD mice. The foremost evidence is provided that hDPSCs promote endogenic neural regeneration by enhancing the activation of the Wnt/β-catenin pathway, which may contribute to stabilizing the hippocampal neural network to reverse memory deficits.

**Conclusion:** These findings highlight the multifunctional potential of hDPSCs in AD treatment, which enhances cognition through alleviating neuropathology and providing neural regenerative driving force. Understanding these multiplicity effects is critical to advancing the clinical translation of stem cell-based therapies for AD.

## Introduction

Alzheimer's disease (AD), the most prevalent age-dependent neurodegenerative disorder, is characterized by progressive cognitive dysfunction concomitant with several typical neuropathological hallmarks: extracellular amyloid-β (Aβ) deposits, intracellular neurofibrillary tangles (NFTs) arising from the accumulation of truncated and hyperphosphorylated tau protein, neuroinflammation, and neuronal loss 1. As a devastating condition with no currently available effective treatments, AD is one of the leading causes of impaired quality of life and mortality among the aging population 2, 3. The costs of health care and long-term care for individuals with AD have been further aggravated by the COVID-19 pandemic 3. Despite recent advancements, current clinical therapies, including symptomatic and disease-modifying approaches, solely mildly alleviate certain symptoms in the early stages of AD and fail to halt disease progression in advanced cases 1, 4. The extremely complicated interactions in the multifactorial pathology of AD and the inability to regenerate lost neurons in the patient's brain pose a great challenge for the pharmaceutical companies. Multiple targets therapies that aim at multiple AD pathologies and neural regeneration are urgently needed to arrest or even cure AD lesions.

Recently, stem cell-based multifaceted approaches have turned out to be endowed with therapeutic potential and translational applications for intractable neurodegenerative disorders. Preclinical studies have demonstrated that transplantation of various stem cell types, including neural stem cells (NSCs), bone marrow-derived mesenchymal stem cells (BM-MSCs), or adipose-derived mesenchymal stem cells (AD-MSCs), which have potent immunomodulatory properties and the ability to secrete multifunctional factors, can significantly enhance cognitive function and modulate neuropathology in various AD models 5-7. Of note, the versatility of stem cell in the treatment of AD is manifested not only in advantageous immunomodulatory and neuroprotection, but also in the unique anti-amyloidogenic activities and differentiation into functional neural cells and integration into the damaged synaptic network in AD brain, thus aiding in the restoration of intracerebral homeostasis in a pleiotropic manner 8. However, the above stem cells still face several challenges in clinical application due to some inherent characteristics, including invasive collection procedures, ethical issues, and limited availability.

Human dental pulp stem cells (hDPSCs), typically derived from freshly extracted impacted third molars, represent a non-invasive alternative for obtaining MSCs that minimizes ethical barriers and expands the available source for clinical applicability 9. The neural crest origin of hDPSCs also endows them with the ability to differentiate into functionally neuron-like cells and oligodendrocyte-like cells in appropriate microenvironmental cues, as well as to secrete some neurotrophic factors 10, 11. The most recent research from our group established several benefits of hDPSCs-based administration for improving the pathophysiological progression of AD 12. We demonstrated that hDPSCs can exert excellent inflammatory control and neuroprotective effects in preclinical models of AD, suggesting that hDPSCs may be a superior source for systematically intervening in cognitive decline. However, whether hDPSCs can directly mitigate neuronal loss, which is a direct participant in cognitive decline, remains unclear. Notably, enhancing neurogenesis in early AD stages has been proposed as a strategy to counteract subsequent neuronal degeneration 13.

Here, we explored the therapeutic efficacy of clinical-grade hDPSCs in promoting adult hippocampal neural regeneration and improving cognitive function in 3xTg-AD mice. Meanwhile, we further revealed that activation of the Wnt/β-catenin signaling pathway may be one of the underlying mechanisms by which hDPSCs rejuvenate endogenic neural regeneration. Collectively, these findings underscore the potential of hDPSCs as a novel intervention strategy for AD.

## Methods

### Clinical-grade hDPSCs and Supernatant fluid collection

Impacted third molars without periodontal disease, periapical disease or caries were obtained from healthy volunteers aged 20-35 years at the Department of Oral Surgery, Renmin Hospital of Wuhan University with ethical approval (Approval Number: WDRY-2022-K025, Wuhan, China). Simply put, freshly extracted teeth were sterilized using 75% ethanol, and the dental pulp tissue was subsequently rinsed three times with phosphate-buffered saline (PBS) and minced into small fragments. Then, the pulp tissue was digested with 4 mg/ml dispase (Sigma, USA) and 3 mg/ml collagenase type I (Gibco, MD) at 37 ℃ for 20 min. Finally, the cellular suspension was seeded in T-25 culture flask at 37 °C in a 5% CO_2_ humidified atmosphere and incubated with Minimum Essential Medium α (α-MEM; Gibco, USA). The culture medium was replaced on day 5 and subsequently refreshed every two days. Supernatant fluid was collected from the culture medium for experiments. The isolation, cultivation, identification, quality control, and storage of hDPSCs were conformed to the quality standards.

### Mice

The 3xTg-AD mice (5-months-old) were kindly gifted from Prof. Wang's lab and the corresponding wildtype (WT) were purchased from the Hubei Provincial Laboratory Animal Public Service Center (Wuhan, China). All procedures performed in studies involving animals were in strict accordance with the US National Institutes of Health Guidelines for the Care and Use of Laboratory Animals and were approved by the Institutional Animal Care and Use Committee (IACUC) of Renmin Hospital of Wuhan University (WDRM2023010D). Mice were maintained in the specific pathogen-free (SPF) conditions with temperature (22 ± 2 °C) and fed with standard pellet diet and pure water. Both male and female mice were used. The animals were divided equally into groups without distinction of sex.

### Genotyping

The offspring of 3xTg-AD mice were genotyped by performing PCR of the genomic DNA clipped from the tails of young mice. The primers for genotyping are listed in Table S2**.**

### Bilateral hippocampal stereotactic injections of hDPSCs

5-month-old mice were anesthetized using 1% pentobarbital sodium (0.1 ml/20 g). The heads of mice were secured in a stereotaxic frame with mouse adaptor (RWD, China). PBS (5 µl) or hDPSCs (5 µl, 1x10^5 cells) were bilaterally injected into the hippocampus at a speed of 100 nl/min with the following coordinates: anteroposterior (AP) -2.06 mm, mediolateral (ML) ± 1.5 mm, dorsoventral (DV) -1.8 mm from bregma. After the injection, the needle was left *in situ* for an additional 5 min before being slowly removed slowly (3min). Post-surgical care was provided, and mice were placed on a heating pad until fully awake.

### Cell proliferative assay

The cytotoxicity of PKH26 (MIDI26, sigma) -labeled hDPSCs or was assessed by cell proliferation assays using Cell Count-8 Kits (CCK-8) assays (Sigma). The cells were plated at a density of 1000 cells per well in 96-well culture plates, with unlabeled cells serving as negative controls. Culture medium containing 10% FBS was used as the blank control. Each condition was replicated across five wells. A 10 µl CKK-8 reagent was added to each well at 0, 1, 2, 3, and 4 days after inoculation, followed by a 2 h incubation at 37 °C. Three group of SH-SY5Y cells (Control group, OA induction and hDPSCs treatment group) were plated at 1000 cells per well in the 96-well culture plates. Cell viability was assessed using CCK-8 assays (Sigma) at 12 h. Last, the absorbance of plates was measured at 450 nm using a microplate reader (EnSight, PerkinElmer, United States).

### Novel object recognition test

Mice were tested in a square open white apparatus (40 × 40 × 40 cm). On day 1, the task started with a 5 min habituation trial in the empty apparatus. On day 2, during the familiarization phase, the mice were again placed in the apparatus containing two identical objects. Exploration was recorded with ANY-maze software in a 5 min trial. On day 3, during the testing phase, one object was replaced with a novel object, and the time spent exploring the two objects was recorded within 5 min. Memory was expressed as a discrimination index (DI), i.e. (seconds on novel)/ (seconds on novel plus seconds on familiar).

### Morris water maze test

The Morris water maze (MWM) test for studying spatial learning and memory in WT, AD mice were performed as described previously 14 with slight modifications. All mice were tested for 5 weeks or 6 months after PBS or hDPSCs transplantation. The MWM (1.2m in diameter) filled with milky water maintained at 22 °C, with an escape platform fixed 1 cm beneath the water surface in the NW quadrant. During the training trails (days 1 to 6), the mice were placed into the water in a random quadrant (SW, SE, or NE) and were allowed 60 s to locate the hidden platform, with 15-min intertrial interval. If mice failed to reach the platform in the allotted time, they were manually guided to it. On day 7, a probe trial without the platform was presented and the percentage of time spent in the quadrant that previously contained the escape platform during task acquisition was measured over 60 s. Latency to find the platform and swim speed were also recorded using video analysis software.

### Fear conditioning test

The ability to form and retain an association in the aversive experience was tested with a standard fear conditioning paradigm that occurs over a period of two days. Mice were placed in the fear conditioning apparatus (7″ W, 7″ D X 12″ H, Sansbio) composed of plexiglass with a metal shock grid floor and allowed to explore the enclosure for 3 min. Following this habituation period, conditioned stimulus consisting of 2 s of a 0.4 mA footshock was presented with a 1 min intertrial interval. One minute following the last stimulation, the mice returned to their home cage. On day 2, the mice underwent a non-shocks situational test, during which mice were placed in the same apparatus used during conditioning on Day 1, and the time of freezing was recorded via a camera and the software provided by Sansbio.

### Quantitative real-time PCR analysis

Total RNA was isolated from the mouse hippocampal tissue using Trizol Reagent. First-strand cDNA was synthesized by incubating 1 µl of total RNA with oligo dT and reverse transcriptase (Superscript III, Invitrogen, Carlsbad, CA, USA), according to the manufacturer's protocol. The primers are listed in Table S2. All qRT-PCR was performed using the Power SYBR Green PCR Master Mix (Life Technologies, Carlsbad, CA, USA) on the Bio-Rad 5-Color System (Bio-Rad, Hercules, CA, USA). GAPDH expression was used as an internal control for normalization. Relative changes in expression levels were calculated using the 2^ -(ΔCT,Tg-ΔCT,control)^ method. All analyses were performed in biological triplicates for each sample.

### Western blotting

Mouse hippocampal tissues were lysed in lysis buffer (50 mM Tris-Hcl, pH 7.4, 40 mM NaCl, 1 mM EDTA, 0.5% Triton X-100, 1.5 mM Na_3_VO_4_, 50 mM NaF, 10 mM sodium pyrophosphate, 10 mM sodium β-glycerophosphate, supplemented with protease inhibitors cocktail). The homogenates were then centrifuged at 12,000 g for 20 min at 4 °C. The clear supernatant was boiled in SDS loading buffer. After SDS-PAGE, the samples were transferred to a PVDF membrane. The membrane was blocked with Tris-buffered saline (TBS) containing 5% nonfat milk and 0.1% Tween 20 (TBST) at room temperature (RT) for 2 h, then incubated with the corresponding primary antibodies overnight at 4 °C, followed by incubation with the secondary antibodies at RT for 1 h, with subsequent detection using ECL substrate. Finally, the membrane was scanned using the Quantity One Imaging system (Bio-Rad, Hercules, CA, USA). The primary antibodies used in this study are listed in Table S3. For the quantifications, band densities were assessed using ImageJ software. The quantification of phosphorylated proteins was normalized to their total proteins. GAPDH was used as the housekeeping protein, and the data were presented as fold change to the control group.

### *In vitro* co-culture experiment

SH-SY5Y and HT22 cells were purchased from ATCC and cultured in six-well plates with DMEM or DMEM/F12 supplemented with 10% FBS and 1% penicillin/streptomycin at a density of 2x10^5^ cells/well. For control group, SH-SY5Y or HT22 cells were cultured with 2 ml complete medium. For okadaic acid (OA, MCE, China) group, HT22 or SH-SY5Y cells were induced by incubation with OA at a final concentration of 20 nm for 24 h as previously reported 15. For co-culture group, a co-culture transwell chamber (0.4 μm pore size; Corning) was used to assess the effects of hDPSCs on OA-damaged HT22 or SH-SY5Y cells. hDPSCs were seeded into the upper compartment at a 1x10^5^ cells/well in 1 mL of α-MEM complete medium with 24 h.

### Nissl staining

Following behavioral testing, mice were perfused with 0.9% saline solution in the left ventricular area, followed by fresh 4% paraformaldehyde (PFA at PH 7.4). After paraffin embedding, three coronal slices were collected from each mouse, containing the dorsal hippocampus at 10 μm thickness. Then, toluidine blue staining (Nissl staining) was performed. Finally, these were dehydrated in absolute ethyl alcohol, cleared in xylene overnight, and mounted with coverslip. The slides were scanned by NanoZoomer, and digital pictures were taken by NDP. View2 software (Hamamatsu, Japan).

### Immunofluorescence

Mice brain tissues were embedded in paraffin, then incubated with Triton X-100 for 10 min and blocked with BSA (0.5%) at RT for 1 h. Tissue samples were then incubated with primary antibodies used in this study are listed in Table S3. After overnight incubation, the samples were incubated with Alexa Fluor® 488-, 555- or 594-conjugated secondary antibodies for 1 h at 37 ℃, counterstained with DAPI, then images were acquired using a fluorescence microscope or a confocal laser scan microscope. The plaque area was calculated using the “analyzed particles” plugin of ImageJ software, and the relative plaque load was determined by dividing the total plaque area by the area of the hippocampus. Phospho-tau immunoreactivity was quantified as the mean signal intensity using ImageJ software. For quantification of Nestin, colocalized β-III Tubulin-NeuN, IBA1, and GFAP-positive cells in hippocampal tissue, cells were manually counted per section using ImageJ software, and the number of positive cells was calculated by averaging the values of three sections.

### Electrophysiology

Mice were anaesthetized with 1% pentobarbital sodium and then decapitated. The brain was removed in ice-cold solution containing 124 mM NaCl, 3 mM KCl, 1.25 mM NaH_2_PO_4_∙2H_2_O, 6.0 mM MgCl_2_, 26 mM NaHCO_3_, 2.0 mM CaCl_2_, and 10 mM D-Glucose. Hippocampus were dissected and cut into 400 mm thick transverse slices with a vibratome. The slices were maintained at room temperature in the solution for at least 60 min. Following incubation, these slices were transferred to a recording chamber on the stage of an up-right microscope and perfused with standard artificial cerebrospinal fluid (a-CSF, 24 °C) at a rate of 2-3 ml/min. For APs recording in the administration site, the grafted GFP cells were identified and recorded under an Olympus upright fluorescence microscope. In current clamp mode, step currents (with steps of 10 pA) were applied to evoke action potentials (APs). For LTP recording in CA1, a 0.1 MU tungsten monopolar electrode was placed to stimulate the Schaffer collaterals pathway. The field excitatory postsynaptic potentials (fEPSPs) were recorded in CA1 stratum radiatum by a glass electrodes filled with a-CSF with resistance of 3-4 MU. The stimulation output was controlled by the trigger function of a multi-clamp 700B amplifier. Data were filtered at 2 kHz, digitally sampled 20 kHz using Pulse software. Synaptic stimulus strengths were measured by generating input-output (I-O) curves for fEPSPs. LTP was induced by 3 theta-burst-stimulation, it is four trains of 100 Hz, repeated 3 times with a 200 ms intervals). The magnitudes of LTP are expressed as the means of baseline fEPSP initial slope. Data was analyzed by GraphPad Prism 9.

### Golgi staining

Mouse brains were fixed in 10% paraformaldehyde for 24 h, and then immersed in 3% potassium bichromate for 3 days away from light. The solution was changed each day. Then the mouse brains were transferred into 2% silver nitrate solution and incubated for 24 h in the dark. Vibratome sections were cut at 60 μm, air dried for 10 min, dehydrated through 95% ethanol and ethanol absolute, cleared in xylene. Bright-field images of pyramidal neurons in the hippocampus were taken at 4X-100X magnification using an Olympus microscope. For the spine density measurements, all clearly evaluable regions of 20-200 μm of secondary dendrites from each imaged neuron were used.

### Primary cultured neurons

Primary mouse cortical neurons were isolated from embryonic day 17 mouse embryos. Cortices were dissociated by 5 ml of D-Hanks containing 0.25% trypsin for 5 min at 37 ℃, centrifuged at 1000 rpm for 5 min after the addition of 4 ml of the neuronal plating medium containing DMEM with 10% fetal bovine serum and 0.5% penicillin-streptomycin. The cells were resuspended, and approximately 4.5x10^5^ cells were plated onto 15 mm poly-L-lysine-coated glass coverslips. The neurons were maintained in serum-free neurobasal medium supplemented with 2% B27 and 1% GlutaMax in a humidified incubator with 5% CO_2_ at 37 °C. The Wnt8b recombinant protein (100 ng/ml) was added to the medium on the third day, and 50% of the medium was changed every 5 days. The primary neurons were collected for relevant experiments on day 7.

### Statistical analysis

All results are presented as the mean ± standard deviation (SD) from three or more independent experiments and analyzed using GraphPad Prism (version 9.0). Statistical analyses were performed using either Student's t-test (two-group comparison) or one-way ANOVA followed by Tukey's or least significant difference (LSD) for post hoc test (more than two groups), and differences with P values less than 0.05 were considered to be statistically significant.

## Results

### Clinical-grade hDPSCs significantly restored spatial learning and memory in 3xTg-AD mice after short-term transplantation

**Figure 1:**
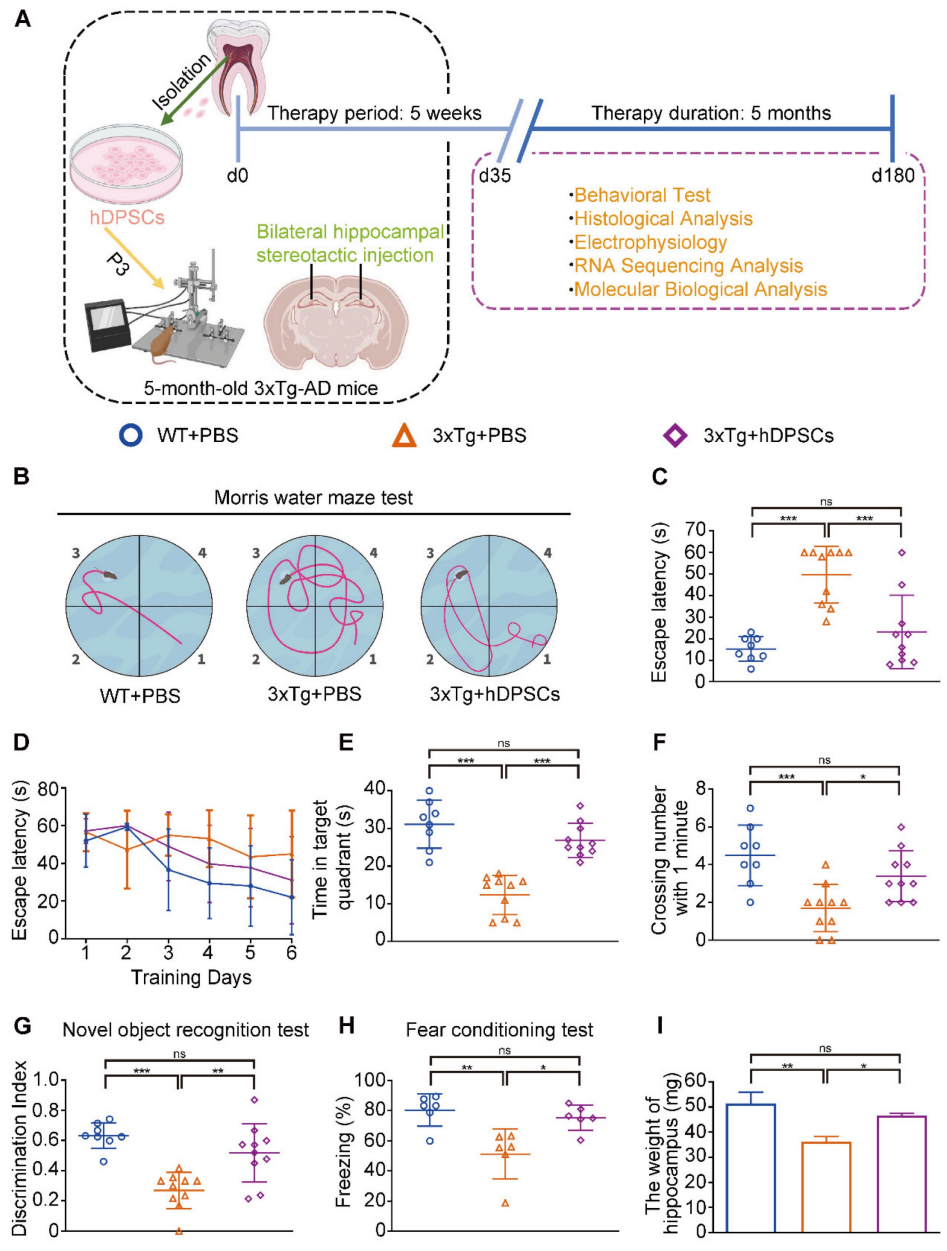
** Clinical-grade hDPSCs significantly restored spatial learning and memory in 3xTg-AD mice after short-term transplantation.** (A) Schematic representation of the experimental design illustrated the chronological order of treatment for 5-month-old 3xTg-AD mice with hDPSCs or PBS by bilateral hippocampal stereotactic single transplantation, followed by behavioral and neuropathological assessments at 5-week (short-term) and 6-month (long-term) treatment points, respectively. (B-F) Morris water maze assays were performed 3xTg-AD mice and the age-matched WT littermates 5 weeks after hDPSCs/PBS therapy. B) The typical escape trajectories, D) the escape latency (s), E) the time mice stayed in the target quadrant without a platform on day 7 and F) the average number of crossings over previous platform location without a platform on day 7 for mice. C) Learning curve showed the average daily escape latency (s) to the hidden platform during the first 6 days acquisition training. (G)The discrimination index (DI) for the novel object recognition test of 3xTg-AD mice and the age-matched WT littermates were recorded after 5 weeks of PBS or hDPSCs treatment. (H) Comparison of the freezing (%) from the three groups of the fear conditioning test. (I) At the end of the behavioral experiment of the 5-week treatment cycle, the hippocampus of partial mice was isolated and weighed. (n = 8-10 mice per group; Values represented mean ± SD, *P < 0.05, **P < 0.01, ***P < 0.001).

To obtain clinical-grade stem cells, hDPSCs were separated and prepared in the good manufacturing practice (GMP) workshop. The primary hDPSCs were expanded to passage 3 and passage 7, and then sent to the National Institutes for Food and Drug Control (NIFDC) for a comprehensive quality test, where all parameters of the stem cell passages met the quality criteria (Table S1). The morphology, phenotype, and differentiation potential of clinical-grade hDPSCs were also characterized (Figure S1A-C). 5 weeks after a single bilateral hippocampal stereotactic transplantation of PKH26-labeled hDPSCs, 3xTg-AD mice were tested in a series of behavioral experiments including the Morris water maze (MWM), the novel object recognition test (NORT), and the fear conditioning test for cognitive function (experimental design in Figure 1A). PKH26-labeled hDPSCs exhibited fibroblast-like with similar morphology and displayed red fluorescence under microscope; the short-term effectiveness of staining was close to 97%, and cell viability remained unchanged (Figure S2A-C). hDPSCs stained with PKH26 were transplanted into the hippocampus of 3xTg-AD mice via stereotactic injection. After 7 days, immunofluorescence revealed the presence of large amounts of red fluorescence-labeled hDPSCs in the hippocampus, which ensured accurate injection (Figure S2D).

In the MWM trial, the 3xTg-AD mice injected with phosphate buffered saline (PBS) (AD+PBS) had longer typical escape paths and escape latency required to reach the hidden platform on day 7 (Figure 1B, C). The age-matched WT mice injected with PBS (WT+PBS) and hDPSCs treated 3xTg-AD mice (AD+DPSCs) showed immediate and orientated. Similarly, the average escape latency of the WT+PBS mice and the AD+DPSCs mice was much shorter than that of the AD+PBS mice on days 3, 4, 5, and 6 (Figure 1D). The swimming time in the target quadrant and the number of times the mice traversed the original platform location within 1 minute were quantified as an index of spatial memory of the platform on day 7; the WT+PBS and AD+DPSCs mice spent significantly more time in the target quadrant and crossed the hidden platforms many times when compared with the AD+PBS mice (Figure 1E, F). As a follow-up test for hippocampal-dependent memory, in the NORT (Figure 1G) and the fear conditioning test (Figure 1H), the time spent exploring new objects and performing non-shock situational tests by the WT+PBS and AD+DPSCs mice was higher than that of the AD+PBS mice, revealing that hDPSCs injection significantly improved memory of the original environment in 3xTg-AD mice. It should be noted that in the operation of separating the hippocampus and cortex among the three groups, significantly less hippocampal weight was found in the AD+PBS mice than that of the WT+PBS mice, and this phenomenon was restored in the AD+DPSCs mice (Figure 1I), while there was no difference in cortical weight (Figure S3A). These results indicated that the single transplantation of hDPSCs significantly restored spatial learning and memory in 3xTg-AD mice in the short term.

### hDPSCs ameliorated neuropathological features in the hippocampus of 3xTg-AD mice

**Figure 2:**
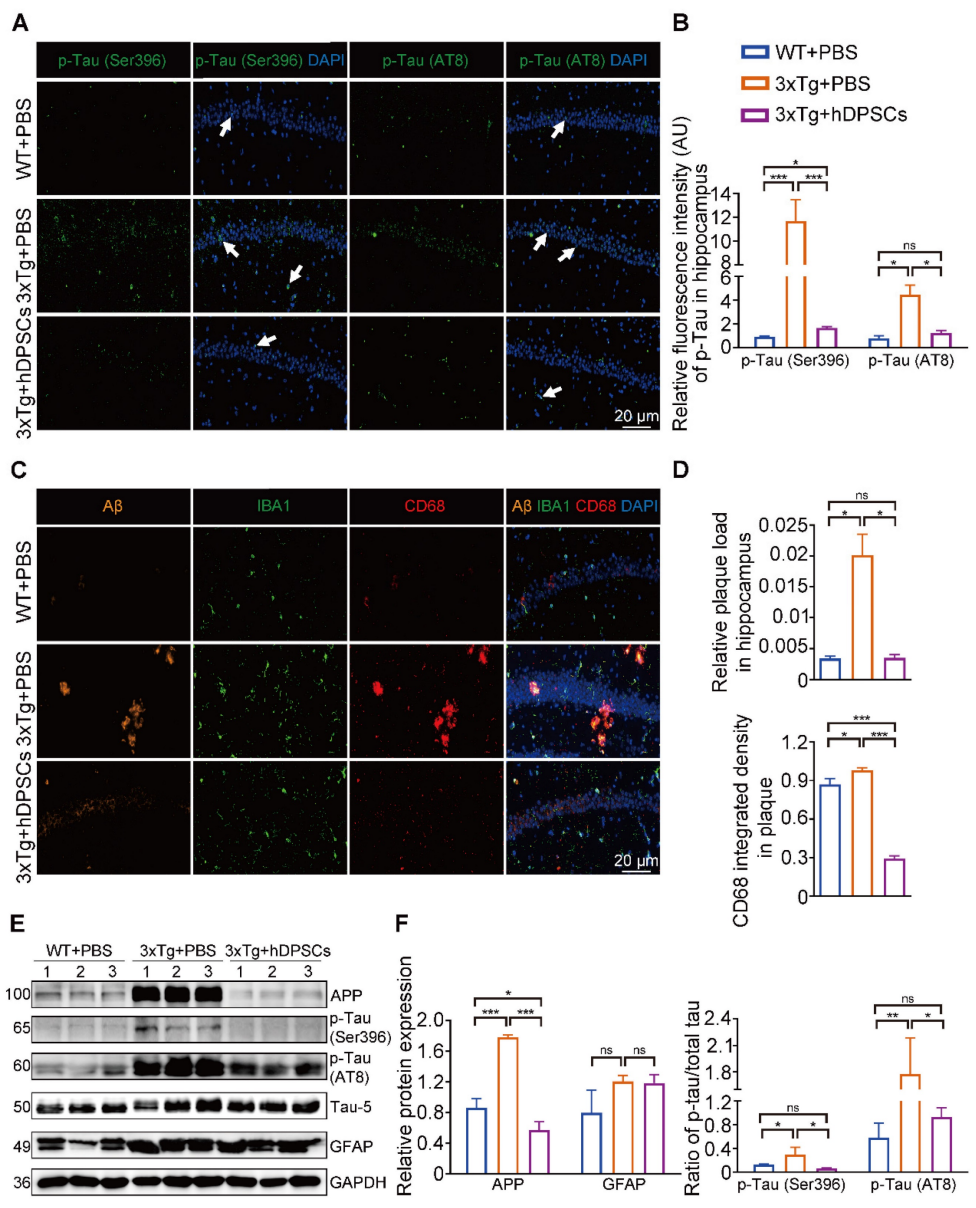
** hDPSCs ameliorated neuropathological features in the hippocampus of 3xTg-AD mice.** (A, B) Representative immunostaining and quantification of p-Tau (Ser396 and AT8, green) in brain sections of the hippocampus from mice 5 weeks after therapy. Scale bar = 20 µm. (C, D) Representative co-immunostaining and quantification of Aβ (orange), IBA1 (green), CD68 (red), and IBA1-CD68 co-localization around plaques (merge) in the hippocampus from mice 5 weeks after therapy. Scale bar = 20 µm. (E) Western blotting results showed the protein expression levels of APP, phospho-Tau (Ser396 and AT8) and GFAP in the hippocampus from three groups mice 5 weeks after therapy. (F) Quantification comparison of protein expression of APP, GFAP, and phospho-Tau (Ser396 and AT8) compared with Tau-5 in the hippocampus, respectively. (n = 3 mice per group; Values represented mean ± SD, *P < 0.05, **P < 0.01, ***P < 0.001).

The hippocampus is a crucial brain region involved in recognition memory mechanisms 16. To determine whether improvements in cognitive performance were accompanied by changes in hippocampal neuropathological features, we analyzed the levels of several crucial pathological targets within the hippocampus of the three groups of mice. Immunofluorescence detection in the hippocampus showed that the relative fluorescence intensity of AD-associated p-Tau (Ser396 and AT8) was significantly decreased in the hDPSCs-treated AD mice (Figure 2A, B and S3B). Staining for amyloid-β (Aβ) plaque showed obviously higher deposition in the hippocampus of AD+PBS mice, which was significantly reduced following hDPSCs-treated AD mice (Figure 2C, D and S3B). Notably, the clearance of pathological deposits of Aβ is often interpreted as depending on the phagocytosis and degradation of activated and aggregated microglia around the plaque 17. We observed that reduced microglia activation occurred in the form of a transformation from a reactive state to a more homeostatic or surveillance state, and a reduction in the CD68-marked area in plaques in the hDPSCs-treated AD mice was readily appreciable as compared to the AD+PBS mice group (Figure 2C, D), implying the ability of hDPSCs administration to convert plaque size and structure to a more indolent state. Consistent with these, western blotting analysis demonstrated that hDPSCs also significantly reduced the expression of APP and p-Tau (Ser396 and AT8) in the hippocampus of AD mice (Figure 2E, F and S3C).

As AD progresses, activated glial cells around plaques have a double-sword action, being neurotoxic in addition to promoting clearance, and proinflammatory cytokines, including interleukin-1β (IL-1β), were found to impair the clearance capacity of glial cells, therefore causing neuronal death 18, 19. We thus assessed the effect of hDPSCs on microglia (IBA1^+^) and astrocyte (GFAP^+^) activation in the mouse brain using immunofluorescence. A significantly increased number of IBA1 and GFAP-labeled cells were found in the hippocampus of AD+PBS mice compared with WT+PBS mice, indicating massive microglia and astrocyte activation and a more pronounced inflammatory response (Figure S4A, B). Likewise, qRT-PCR results showed that the mRNA expression levels of the two most representative proinflammatory cytokines of AD 20, IL-1β and tumor necrosis factor-α (TNF-α), were significantly increased in the hippocampus of AD+PBS mice (Figure S4C). For comparison, hDPSCs-treated AD mice showed a marked reduction in positive numbers in IBA1 and GFAP-labeled cells in the hippocampus and significantly increased expression of anti-inflammatory cytokines. These data suggested that a single administration of hDPSCs is sufficient to decrease amyloidosis, tau hyperphosphorylation, and neuroinflammation in the hippocampus of 3xTg-AD mice in the short term.

### hDPSCs effectively alleviated AD pathologically induced neural cell damage *in vitro*

**Figure 3:**
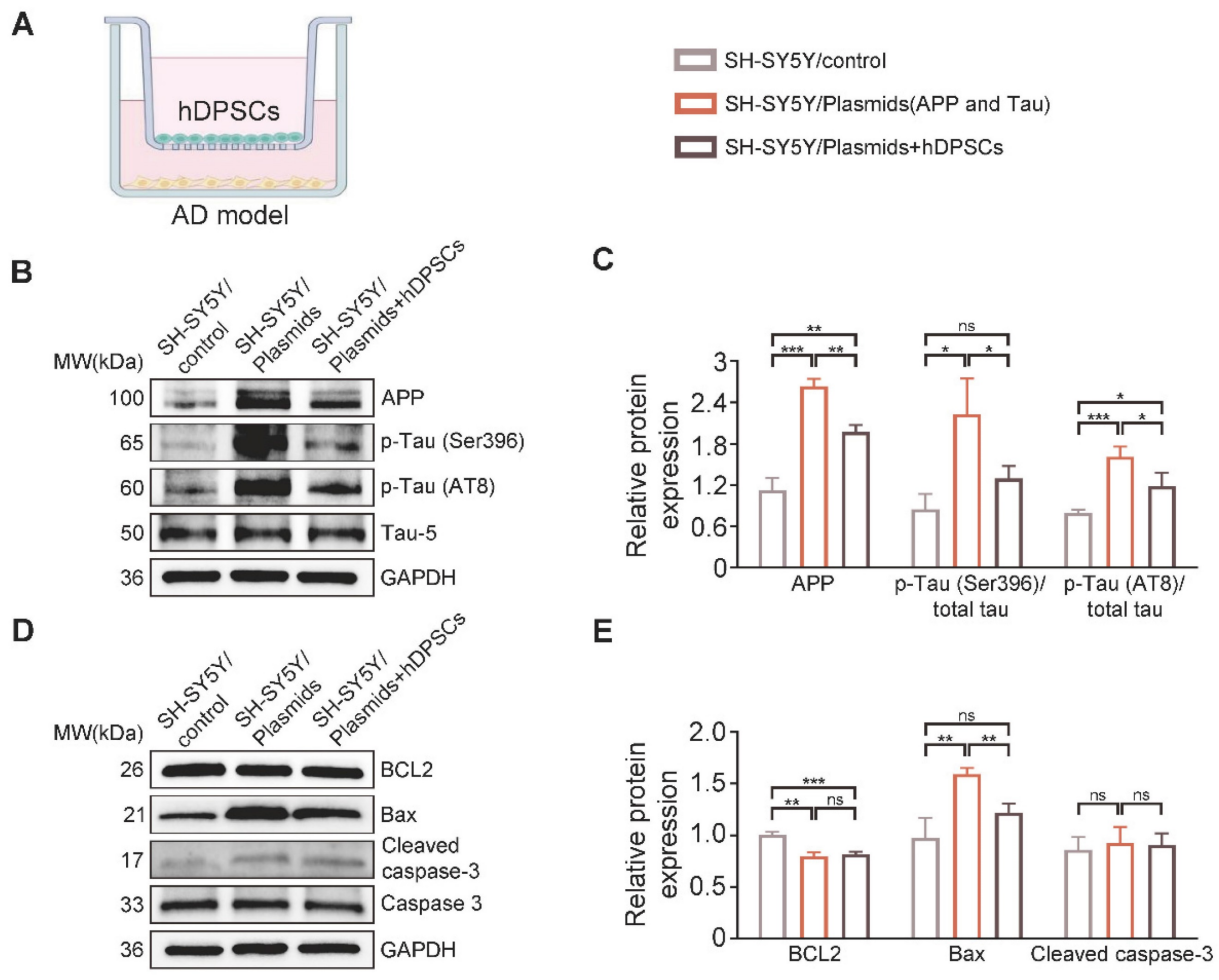
** hDPSCs effectively alleviated AD related plasmids-transfected SH-SY5Y cells damage *in vitro*.** (A) A transwell non-contact coculture assay system was used with hDPSCs and the AD model. (B, C) The protein expression levels and quantification of APP and phospho-Tau (Ser396 and AT8) in the APP and Tau- transfected SH-SY5Y cells of the AD model were tested by western blotting. (D, E) The expression levels and quantification of apoptosis-associated proteins BCL2, Bax, caspase3, and cleaved caspase-3 in the APP and Tau- transfected SH-SY5Y cells of the AD model were tested by western blotting. (n = 3 per group; Values represented mean ± SD, *P < 0.05, **P < 0.01, ***P < 0.001).

Next, to further confirm whether hDPSCs could modulate AD pathology in an *in vitro* AD model, we transfected SH-SY5Y cells 48 h with plasmids expressing the same mutant forms of APP and Tau as those in 3xTg-AD mice to simulate the two major pathological characteristics within AD, followed by co-culturing with hDPSCs for 24 h (Figure 3A). Western blots results revealed that the protein expression of APP and p-Tau (Ser396, AT8) in SH-SY5Y cells after co-culture of hDPSCs was significantly decreased compared with that of the non-co-cultured SH-SY5Y cells, which was consistent with the results observed *in vivo* (Figure 3B, C). The results of apoptosis proteins further characterize this reparative effect, i.e., hDPSCs prevent the imminent apoptosis triggered by AD pathology (Figure 3D, E).

Meanwhile, to further demonstrate the paracrine efficacy of hDPSCs, we also established an *in vitro* therapeutic model in which hDPSCs were co-cultured with SH-SY5Y cells induced by the classical tau phosphorylation inducer okadaic acid (OA). As previously described 15, SH-SY5Y cells were incubated with OA at a final concentration of 20 nmol/ml in the lower compartment for 24 h, and then hDPSCs were inoculated in the upper compartment for 24 h to establish *in vitro* co-culture model. Next, we analyzed cell morphology under light microscopy, levels of tau phosphorylation, mitochondrial function and structure, and cell viability to assess the extent of OA-induced damage and the therapeutic effect of hDPSCs. As shown in Figure S5A, the normal SH-SY5Y cells observed under light microscopy are shuttle-shaped or polygonal with elongated dendrites characteristic of neuronal cells; after exposure to OA, most cells retracted into a rounded shape, decreasing in number. Contrary to that, the cell morphology was restored, and cell density and viability were distinctly increased in the hDPSCs treatment group (Figure S5A, B). Based on the characteristics of tau hyperphosphorylation, we examined tau phosphorylation levels in OA-induced SH-SY5Y cells by western blotting and found that hDPSCs could effectively inhibit tau hyperphosphorylation (Figure S5C, D). Similarly, we found that hDPSCs treatment group significantly decreased the expression of apoptotic proteins, cleaved caspase-3 and Bax, and increased the expression of the anti-apoptotic protein BCL2 in OA-induced SH-SY5Y cells (Figure S5E, F). Subsequent investigations with HT22 mouse hippocampal cells also show consistent results (Figure S6A-D).

Mitochondria play a critical role in cellular energy production and apoptosis, and disturbances of mitochondrial function and structure are closely associated with the pathogenesis of AD 21. The results of mitochondrial membrane potential (MMP) reflecting early apoptosis of cells showed that hDPSCs clearly inhibited OA-induced MMP depolarization in SH-SY5Y cells (Figure S5G, H). A further observation of mitochondrial ultrastructure under transmission electron microscopy (TEM) showed that the mitochondria of OA-induced SH-SY5Y cells presented collapse, swelling, and a disorderly arrangement of mitochondrial cristae. While in the hDPSCs treatment group, the intracellular mitochondria returned to a regular shape, and mitochondrial cristae were arranged in an orderly manner, basically similar to that in the control group (Figure S5I). These studies collectively indicated that hDPSCs can effectively alleviate AD pathologically induced neural cell damage *in vitro*.

### hDPSCs rejuvenated endogenous neural regeneration in the hippocampus of 3xTg-AD mice

**Figure 4:**
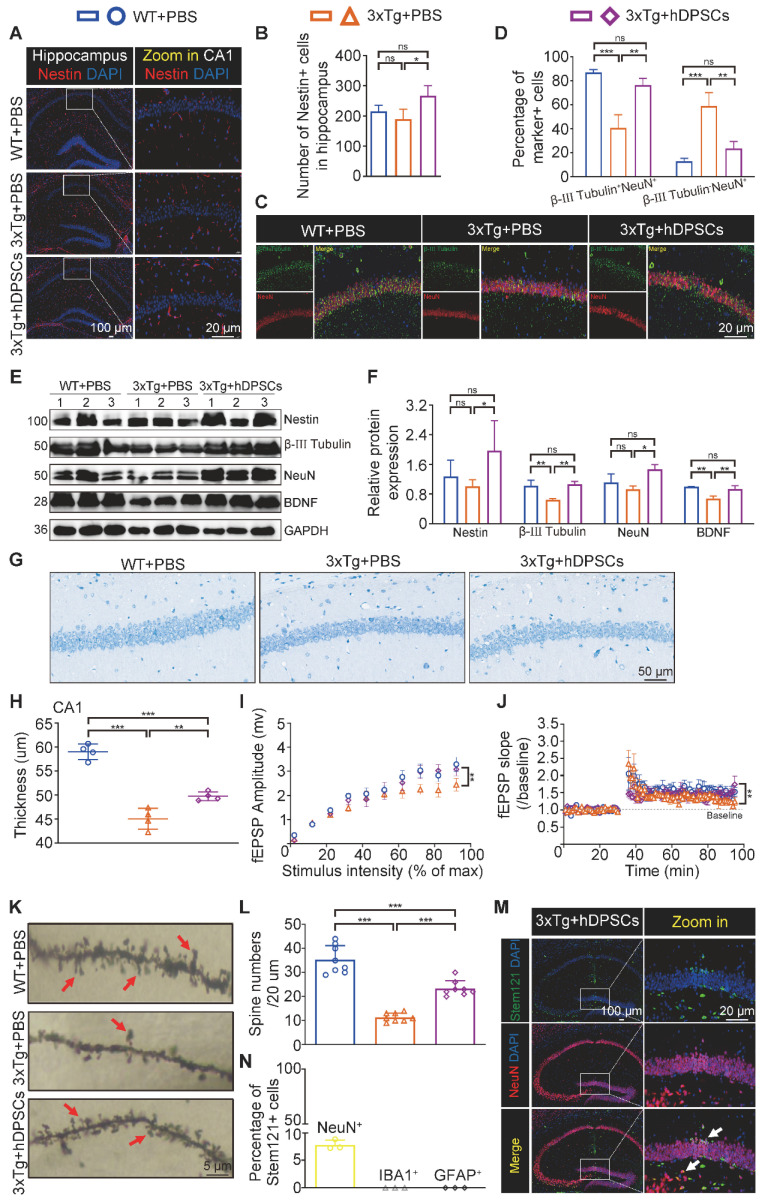
** hDPSCs rejuvenated endogenous neural regeneration in the hippocampus of 3xTg-AD mice.** (A, B) Representative immunostaining and quantification of Nestin (red)-positive cells in brain sections of the hippocampus from mice 5 weeks after therapy. Scale bar = 100 µm, 20 µm. (C) Representative co-immunostaining of β-III Tubulin (green) and NeuN (red) in brain sections of the hippocampus (CA1) from mice 5 weeks after therapy. (D) Shown in quantification is a summary of the distribution of neurons at different maturation stages. Scale bar = 20 µm. (E, F) Western blotting results showed the protein expression levels and quantification of Nestin, β-III Tubulin, NeuN and BDNF in the hippocampus from mice 5 weeks after therapy. (G) Representative Nissl-stained neurons in the CA1 region of the hippocampus from mice 5 weeks after therapy. Scale bar = 50 µm. (H) Quantification of the cell layer thickness. (I, J) Representative traces showed input-output curves and LTP of fEPSPs in CA1 of WT and 3xTg-AD mice treated with PBS or hDPSCs. (K, L) Representative Golgi staining and quantification of spine density (red arrows) in the hippocampus from mice 5 weeks after therapy. Scale bar = 5 μm. (M) Representative immunostaining of Stem121 (green) and NeuN(red) to detect the differentiation status of hDPSCs in brain sections of the hippocampus from mice 5 weeks after therapy. Scale bar = 100 μm (main images), 20 µm (magnified images, white arrows). (N) Quantification of the percentage of Stem121^+^NeuN^+^ neurons in all hippocampal neurons (n = 3 mice per group; Values represented mean ± SD, *P < 0.05, **P < 0.01, ***P < 0.001).

Impaired memory and cognitive dysfunction in humans and rodents with AD are increasingly being linked to impeded adult hippocampal neurogenesis 22. After clarifying the ability of hDPSCs to efficiently alleviate neuropathological burden, we further evaluated the potential role of hDPSCs in the fate of neurons within the adult hippocampus of 3xTg-AD mice. As expected, immunofluorescent analysis of the neural stem cell marker Nestin revealed a significant increase in the number and viability of Nestin^+^-labeled cells (NSCs) in the hippocampus of hDPSCs-treated AD mice, compared with that in the AD+PBS mice (Figure 4A, B). To dissect whether hDPSCs also modulate newborn neuron maturation, which is concurrent with ongoing neuronal circuit activity, we analyzed the co-localization of the immature neuronal marker β-III Tubulin and the mature neuronal marker NeuN in the hippocampus. A substantial decrease in the percentage of β-III Tubulin^+^NeuN^+^-labeled neurons was observed in the CA1 region of AD+PBS mice compared with age-matched WT mice and hDPSCs-treated mice, suggesting that the presence of hDPSCs rejuvenated the maturation of immature neurons in the adult hippocampus of 3xTg-AD mice, thereby alleviating the neuronal damage under pathological conditions of AD (Figure 4C, D). However, the 5-weeks therapeutic effect of hDPSCs did not seem to affect the maturation process of neurons in the dentate gyrus (Figure S7A, B). Subsequent protein expression assays of above neural markers corroborated these results (Figure 4E, F). Interestingly, the existence of hDPSCs also induced the elevation of brain-derived neurotrophic factor (BDNF), which has emerged as an essential neurotrophin for adult hippocampal neurogenesis and the maintenance of cognitive intactness in an AD mouse model 13.

The impressive neuroprotective action of hDPSCs treatment is also reflected in the results of Nissl-stained brain slices from AD mice. The thickness of the neural cell layer measured in the CA1 region showed that the cell layer thickness of AD+PBS mice was significantly thinner than that of WT mice, whereas after treatment with hDPSCs, neural cell layer thickness increased significantly (Figure 4G, H). Considering the reduced hippocampal weight in AD+PBS mice (Figure 1I), the results of hippocampal area 23 and systematically measuring the distance between the CA1 and DG regions at three different levels (D1, D2, and D3) as a quantitative criterion for hippocampal atrophy 20 showed that hippocampal atrophy was more pronounced in AD+PBS mice, whereas it was alleviated in the hDPSCs-treated mice (Figure S8A, B), which was further consolidated by Tunel staining as demonstrated in Figure S9A. This is consistent with the results of apoptosis-associated protein assays in hippocampal tissue. After 5 weeks of hDPSCs treatment, the expression of pro-apoptosis protein cleaved caspase-3 and Bax was significantly decreased in hippocampal tissue of AD mice, while the expression of anti-apoptosis protein BCL2 was elevated (Figure S9C, D). The nature of cell apoptosis was also confirmed by immunofluorescence staining of cleaved caspase-3 (Figure S9B). Further, we demonstrated that hDPSCs significantly improved the long-term potentiation (LTP) in the CA1 area of 3xTg-AD mice and enhanced hippocampal synaptic plasticity (Figure 4I, J). Golgi staining also showed relatively obvious increases in both the branch number of dendritic spines along individual dendrites of CA1 region neurons and the neuronal density of the DG region in the hDPSCs-treated mice compared to that of the AD+PBS mice (Figure 4K, L and S10). These resulted suggested that hDPSCs promoted neuronal protective mechanisms, together with lowered apoptosis in 3xTg-AD mice, which could at least partially explain the increase in the number of neurons we observed.

Notably, given the ability of cranial neural crest-derived hDPSCs to differentiate into functional neuron-like cells, which may also be closely coupled with the regeneration of the lost neurons, the transplanted hDPSCs and their differentiation status in the hippocampus of AD mice were detected by double immunofluorescence staining for the specific anti-human cell marker Stem121 and neural markers NeuN, IBA1, and GFAP. At 5 weeks after transplantation, abundant double-labeled Stem121^+^NeuN^+^ neurons were clearly observed around the stereotaxic injected site in the hippocampus, especially in the DG region, with scattered positive expression neurons in other regions of the hippocampus (Figure 4M). The percentage of Stem121^+^NeuN^+^ neurons was approximately 7.8% of all hippocampal neurons, indicating that a portion of hDPSCs transplanted into the hippocampus of AD mice were capable of spontaneously differentiating into newborn neurons (Figure 4N). What is mind-boggling is that the differentiation of hDPSCs to microglial cells or astrocytes was very little (Figure S11A, B). Further analysis showed that the transplanted hDPSCs predominantly gave rise to GAD67^+^ GABAergic neurons as well as a few ChAT^+^ cholinergic neurons, and no VGlut1^+^ glutamatergic neurons were detected (Figure S12A-C).

We then examined whether transplanted hDPSCs-derived neurons became functional. To this end, hDPSCs labeled with GFP were bilaterally transplanted into the hippocampus, and whole-cell records were performed at the time of transplantation and 5 weeks after transplantation. GFP-positive cells in acute slices were identified under a fluorescence microscope (Figure S13A). Unfortunately, due to the rapid fluorescence quenching, we were unable to derive an effective fluorescence image. Larger current injections in the GFP-positive neurons recorded could generate action potential (AP), and most of them discharged repetitively without failure (Figure S13B-D), indicating that hDPSCs differentiation into newborn neurons mature in the host hippocampus and acquire functional properties around 5 weeks after transplantation. Together, these phenomena suggested that hDPSCs promoted endogenous neural survival and regeneration by multiple forms of cell-autonomous manner in the adult hippocampus.

### hDPSCs regulating adult hippocampal neural regeneration via activation of canonical Wnt/β-Catenin pathway

**Figure 5:**
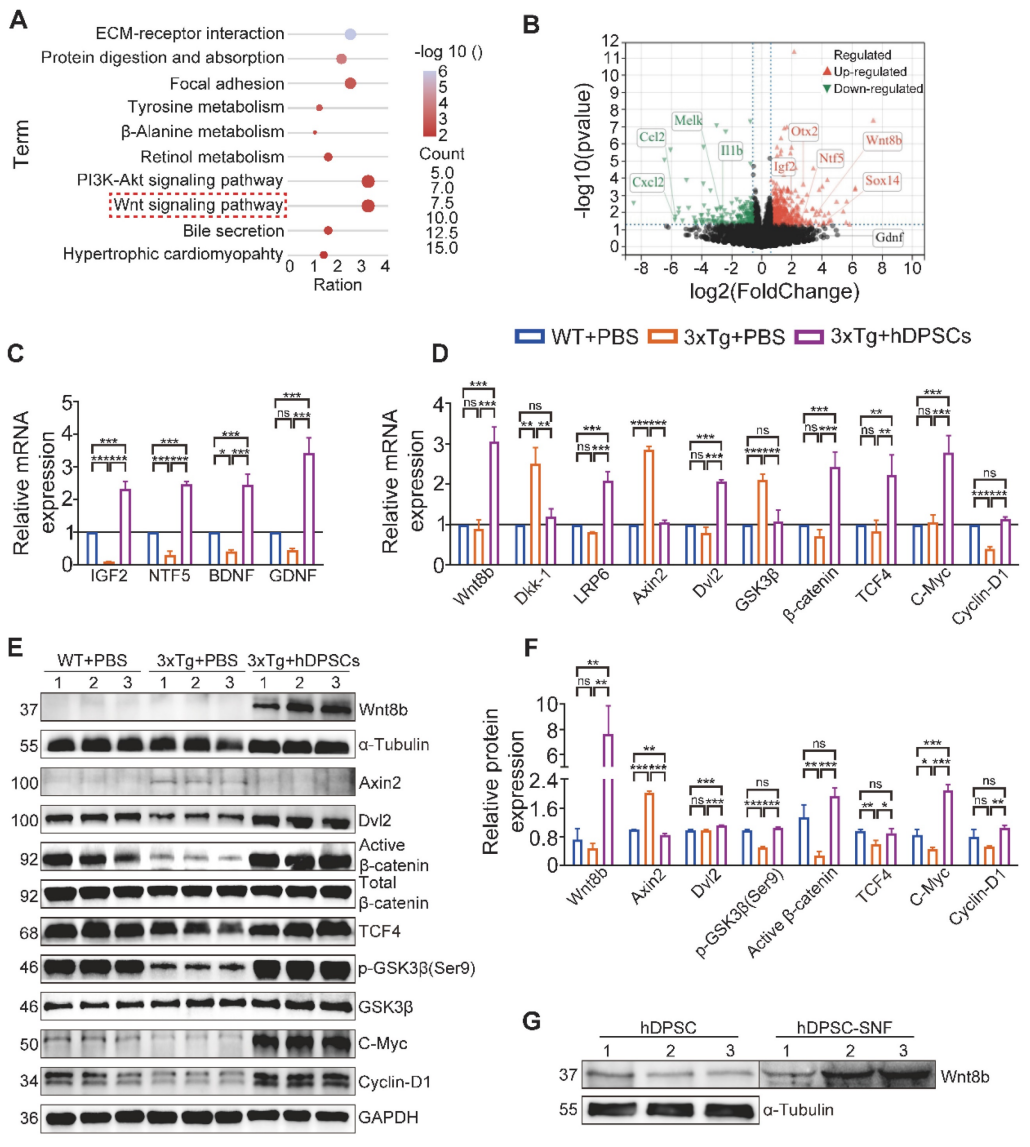
** hDPSCs regulating adult hippocampal neural regeneration via activation of Canonical Wnt/β-Catenin Pathway.** (A) RNA-Seq and the top 10 enriched Kyoto Encyclopedia of Genes and Genomes (KEGG) pathway terms of differentially expressed genes (DEGs) between hDPSCs treatment group and PBS control group in 3xTg-AD mice. (B) Volcano map of differentially expressed genes (DEGs) between hDPSCs treatment group and PBS control group in 3xTg-AD mice. (C) Quantitative real-time PCR analysis was performed for the relative mRNA expression of trophic factor genes in the hippocampus from mice 5 weeks after therapy. (D) Quantitative real-time PCR analysis was performed for the relative mRNA expression of canonical Wnt pathway targets in the hippocampus from mice 5 weeks after therapy. (E, F) The protein expression levels and quantification of canonical Wnt pathway targets in the hippocampus from mice 5 weeks after therapy were tested by western blotting. (G) Western blotting results showed the protein expression levels of Wnt8b from three different hDPSCs and their unconcentrated supernatant fluid. (n = 3 mice per group; Values represented mean ± SD, *P < 0.05, **P < 0.01, ***P < 0.001).

To better understand the possible pathways mediating hDPSCs to promote neural regeneration, we performed RNA-sequencing (RNA-seq) analysis of hippocampus tissue derived from the three groups of experimental mice. The Kyoto Encyclopedia of Genes and Genomes (KEGG) pathway analysis was carried out on all screened differentially expressed genes (DEGs) between the AD+PBS and hDPSCs-treated group, and the top 10 enriched pathways are shown in Figure 5A. Upon comparison of the candidate pathways, the Wnt signaling pathway and the PI3K-Akt signaling pathway with the highest enrichment ratio attracted our attention. The results of Gene Set Enrichment Analysis (GSEA) are also consistent with these (Figure S14A-C). Among them, the Wnt/β-catenin signaling pathway has been shown to control the survival, proliferation, and differentiation of NSC and regulate microenvironment homeostasis in the adult brain 24, 25. As important extracellular secreted glycoproteins involved in regulating adult hippocampal neurogenesis, Wnt ligands also play a specific role in the pathophysiological progression of neurodegenerative diseases such as Alzheimer's and Parkinson's disease 26, 27. Further Gene Ontology (GO) analysis revealed that the Wnt8b gene in the Wnt signaling pathway was involved in the biological process of neuron differentiation and was upregulated in the volcano plots of DEGs between the hDPSCs-treated group versus AD+PBS group (Figure 5B, S15). Therefore, these bioinformatics analysis data pointed out that Wnt8b may be one of the activity-dependent intrinsic regulators of hDPSCs enhancement of adult hippocampal neurogenesis.

Accumulating evidence suggests that several Wnt proteins, including Wnt3a, Wnt7a and Wnt8b, modulate the generation of neurons in the DG region and synaptic transmission in mature vertebrate hippocampal neurons 26, 28, 29. β-catenin, another key component of the Wnt signaling pathway, also plays a central role in the regulation of neural crest cell lineage relationships and neural progenitor cell proliferation in the nervous system 30. To validate RNA-seq results and explore the expression levels of Wnt ligands and downstream molecules at the mRNA and protein levels, hippocampal tissue from three groups of mice was examined by qRT-PCR. Expression of factors associated with pathological improvement in AD, including BDNF, was markedly elevated in the hippocampus of hDPSCs-treated mice compared to the AD+PBS mice (Figure 5C). Simultaneously, compared to the AD+PBS mice, hDPSCs significantly enhanced the mRNA and protein expression of Wnt8b, β-catenin and their downstream molecules (TCF4, c-Myc, and Cyclin-D1), and decreased the expression of β-catenin destruction complex (Axin2 and GSK-3β) in the hippocampus (Figure 5D-F). Indeed, Lako *et al.* have shown that in human and mouse patterns, Wnt8b expression was precisely restricted to the developing forebrain and might play an important role in coordinating cell proliferation and whole neurogenesis within the hippocampus 31. From a developmental perspective, enhanced Wnt8b signaling recruits the formation of the β-catenin/TCF4 complexes, which was needed for the maintenance of early neural differentiation and forebrain developmental specification 32, 33. Further, protein detection of stem cells and their unconcentrated supernatant fluid (SNF) indicated that neural crest-derived hDPSCs could express and secrete Wnt8b (Figure 5G). Meanwhile, we compared the expression of Wnt8b in hDPSCs and human umbilical cord mesenchymal stem cells (hUC-MSCs) exposed to brain homogenate from 3xTg-AD mice at different ages. Wnt8b is expressed specifically in hDPSCs, whereas it is barely expressed in hUC-MSCs, which may be a specifically potential pathway for hDPSCs to mediate neural regeneration and cognitive improvement in AD mice via the Wnt8b/β-catenin pathway (Figure S14D).

In view of the multiple different types of cells in the hippocampal tissue, we next sought to determine whether Wnt8b would have a specific impact on neuronal development. The cytoskeleton of primary mouse neurons was labeled with multiple fluorescence, and then the analysis of dendrites revealed that the increased number of branches in Wnt8b-treated primary neurons (Figure S16A, B). We also investigated the level of related proteins in the Wnt8b-treated primary neurons. The results indicated that Wnt8b promoted the maturation of primary mouse neurons by activating the canonical wnt/β-catenin signaling pathway (Figure S16C-F). Therefore, these findings suggest that the activated Wnt8b/β-catenin signaling pathway may be one of the mediators by which hDPSCs enhance adult hippocampal neural regeneration and are essential for the structural recovery of damaged hippocampal neural networks.

### A single intracerebral treatment with hDPSCs attenuated cognitive deficits and neuropathological progression in 3xTg-AD mice in the long term

**Figure 6:**
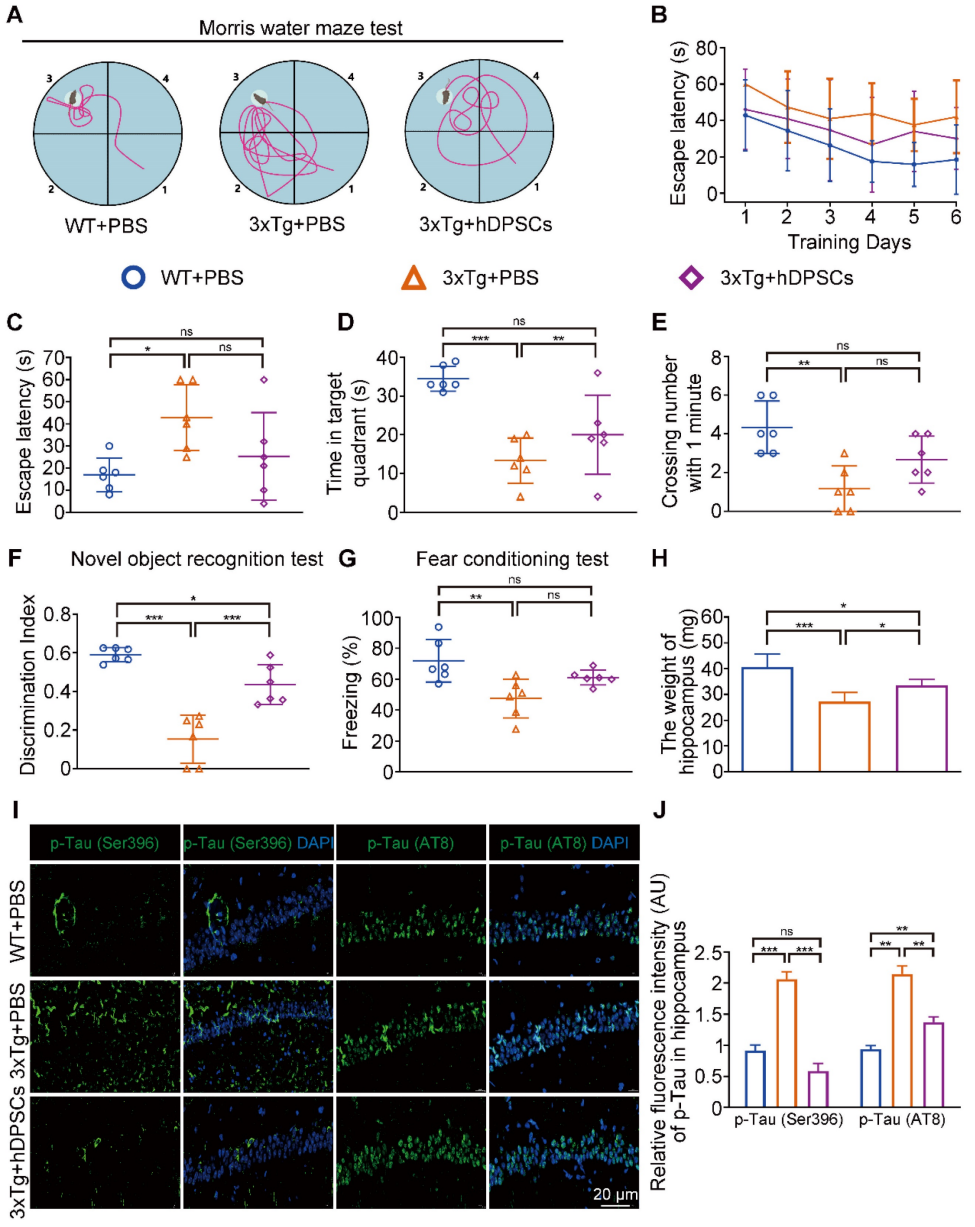
** A single intracerebral treatment with hDPSCs attenuated cognitive deficits and neuropathological progression in 3xTg-AD mice in the long term.** (A-E): Morris water maze assays were performed 3xTg-AD mice and the age-matched WT littermates 6 months after hDPSCs/PBS therapy. A) The typical escape trajectories, C) the escape latency (s), D) the time mice stayed in the target quadrant without a platform on day 7 and E) the average number of crossings over previous platform location without a platform on day 7 for mice. B) Learning curve shows the average daily escape latency (s) to the hidden platform during the first 6 days acquisition training. (F) The discrimination index (DI) for the novel object recognition test of 3xTg-AD mice and the age-matched WT littermates were recorded after 6 months of PBS or hDPSCs treatment. (G) Comparison of the freezing (%) from the three groups of the fear conditioning test. (H) At the end of the behavioral experiment of the 6 months treatment cycle, the hippocampus of partial mice was isolated and weighed. (I, J) Representative immunostaining and quantification of p-Tau (Ser396 and AT8, green) in brain sections of the hippocampus from mice 6 months after therapy. Scale bar = 20 µm. (n = 3-6 mice per group; all data shown as mean ± SD, *P < 0.05, **P < 0.01, ***P < 0.001).

Hitherto, most studies on MSCs for AD treatment have been limited to assessing short-term efficacy after a single injection, whereas long-term efficacy remains unknown 15, 34. The above results prompted us to decipher whether a single intracerebral treatment with hDPSCs of specific origin further mediated long-term therapeutic efficacy in 3xTg-AD mice. As shown in Figure 6A-E, 6 months after transplantation, hDPSCs-treated mice still exhibited relatively better cognitive function, with shorter swimming paths and longer stays in the target quadrant during the MWM task relative to AD+PBS mice. Although not statistically different, hDPSCs-treated mice had a shorter mean escape latency and more frequent hidden platform traversing than did AD+PBS mice during day 7 (Figure 6C, E). In the NORT (Figure 6F) and the fear conditioning test (Figure 6G), all three groups of mice showed a slight decrease in the discrimination index and the freezing time relative to the short-term therapeutic efficacy of 5 weeks, which may be due to aging and disease progression, but cognitive deficits were attenuated in hDPSCs-treated mice compared to the AD+PBS mice. Similar phenomena were observed for changes in hippocampal weight in mice. The hippocampal weight of hDPSCs-treated mice was only slightly, albeit statistically significantly, higher than that of AD+PBS mice (Figure 6H).

**Figure 7:**
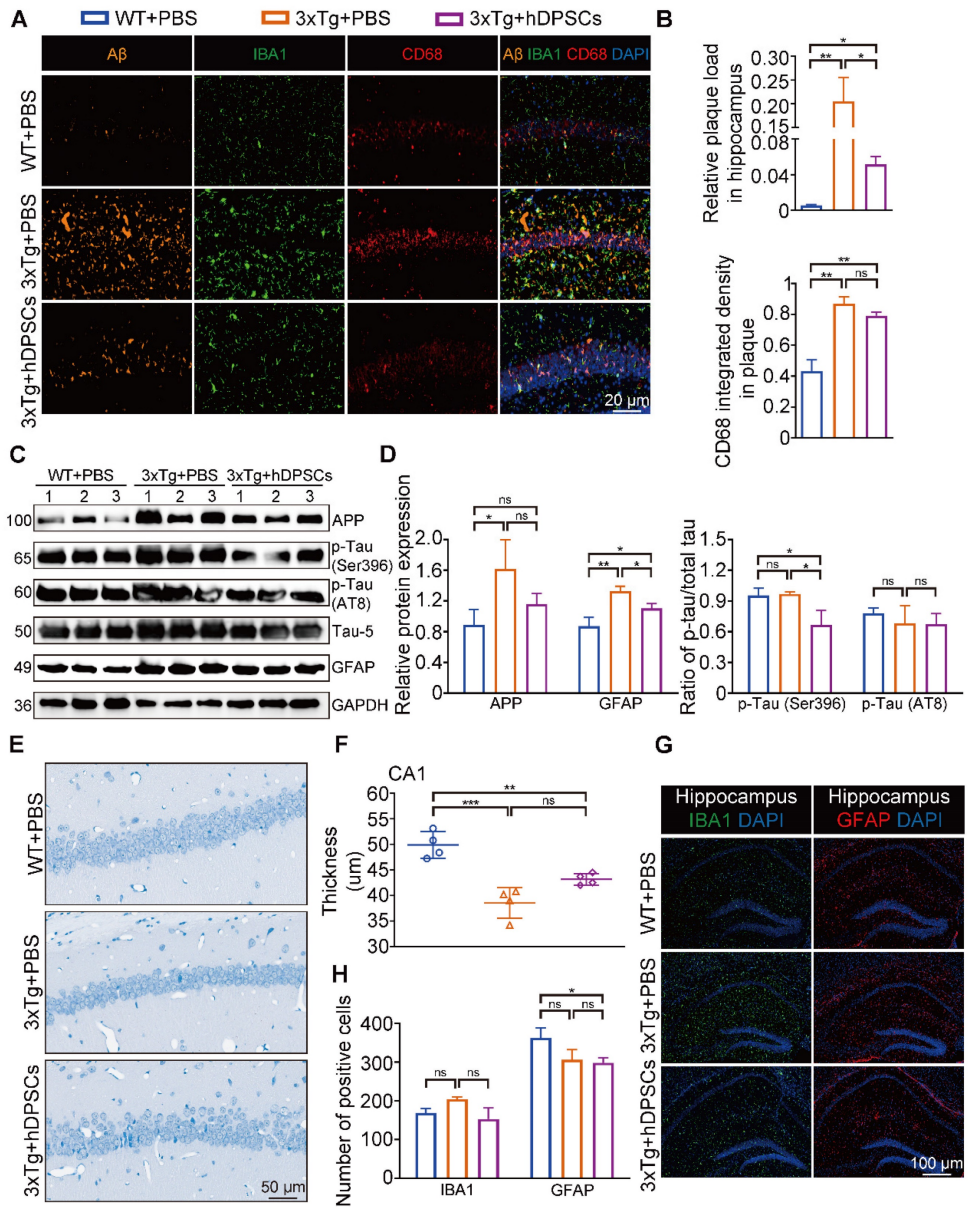
** A single intracerebral treatment with hDPSCs attenuated cognitive deficits and neuropathological progression in 3xTg-AD mice in the long term.** (A, B) Representative co-immunostaining and quantification of Aβ (orange), IBA1 (green), CD68 (red), and IBA1-CD68 co-localization around plaques (merge) in the hippocampus from mice 6 months after therapy. Scale bar = 20 µm. (C) The protein expression levels of APP, phospho-Tau (Ser396 and AT8) and GFAP in the hippocampus from mice 6 months after therapy were tested by western blotting. (D) Quantification of APP, GFAP and phospho-Tau (Ser396 and AT8) compared with Tau-5 in the hippocampus, respectively. (E) Representative Nissl-stained neurons in the CA1 region of the hippocampus from mice 6 months after therapy. Scale bar = 50 µm. (F) Quantification of the cell layer thickness. (G) IBA1 and GFAP immunostaining showing the activation extent of gliosis in the hippocampus from mice 6 months after therapy. Scale bar = 100 µm. (H) Quantification of the IBA1- and GFAP-marked cells in the hippocampus, respectively. (n = 3-4 mice per group; all data shown as mean ± SD, *P < 0.05, **P < 0.01, ***P < 0.001).

After behavioral tests, we further explored the effects of hDPSCs on AD pathology in the long term. Surprisingly, fluorescence analysis showed a significant reduction in the relative fluorescence intensity of p-Tau (Ser396 and AT8) in the hippocampus of the hDPSCs-treated mice compared to AD+PBS mice; the relative fluorescence expression of p-Tau (Ser396) in the hippocampus of hDPSCs-treated mice was even lower than that of WT mice (Figure 6I, J and S17). Aβ accumulates in the brain as the disease progresses, which in turn induces more aberrant neural circuit activity. Again, immunofluorescence staining for amyloid plaques on brain tissue from 3xTg-AD mice treated with hDPSCs showed a visible reduction in the plaque load of hippocampal Aβ plaques (Figure 7A, B and S17). A co-localization of plaques, microglia, and CD68 well revealed that hDPSCs-treated mice appeared to have a slight decrease in the integrated density of the CD68-marked area surrounding the plaques relative to AD+PBS mice (Figure 7A, B). The results were also supported by the detection of protein expression related to markers in the hippocampus (Figure 7C, D). We then assessed the overall level of neuroinflammatory in the hippocampus of AD mice at this stage. Quantitative IBA1- and GFAP-positive cell analysis by immunofluorescence corroborated that both the number of microglia and astrocyte activation were reduced in the hDPSCs-treated mice as compared to those treated with PBS, albeit not statistically significant (Figure 7G, H). Furthermore, to investigate whether a single transplantation of hDPSCs protects against neuronal cell death induced by the pathological progression of AD in the long term, Nissl staining and Tunel staining was performed and quantified. The results manifested that although there was no statistically significant difference, the neuronal layer thickness in the CA1 region was still greater in the hDPSCs-treated mice than in the AD+PBS mice (Figure 7E, F); the pathological progression of AD exacerbated neural cell apoptosis, which was alleviated by transplantation of hDPSCs (Figure S18A). The western blotting analysis also confirmed that, although there were no rules to follow for Bax expression, cleaved caspase-3 expression was significantly downregulated and BCL2 expression was increased in hDPSCs-treated mice as compared with that in AD+PBS mice (Figure S18C, D), which was further identified by immunofluorescent staining of cleaved caspase-3 (Figure S18B). Collectively, these data all supported the fact that a single administration with hDPSCs in 3xTg-AD mice still achieves better cognitive and partially subregional neuropathological improvements over the long term (6 months), although not as significant as the short-term efficacy (5 weeks).

### hDPSCs partially imparted sustained hippocampal neural regeneration in 3xTg-AD mice over time

**Figure 8:**
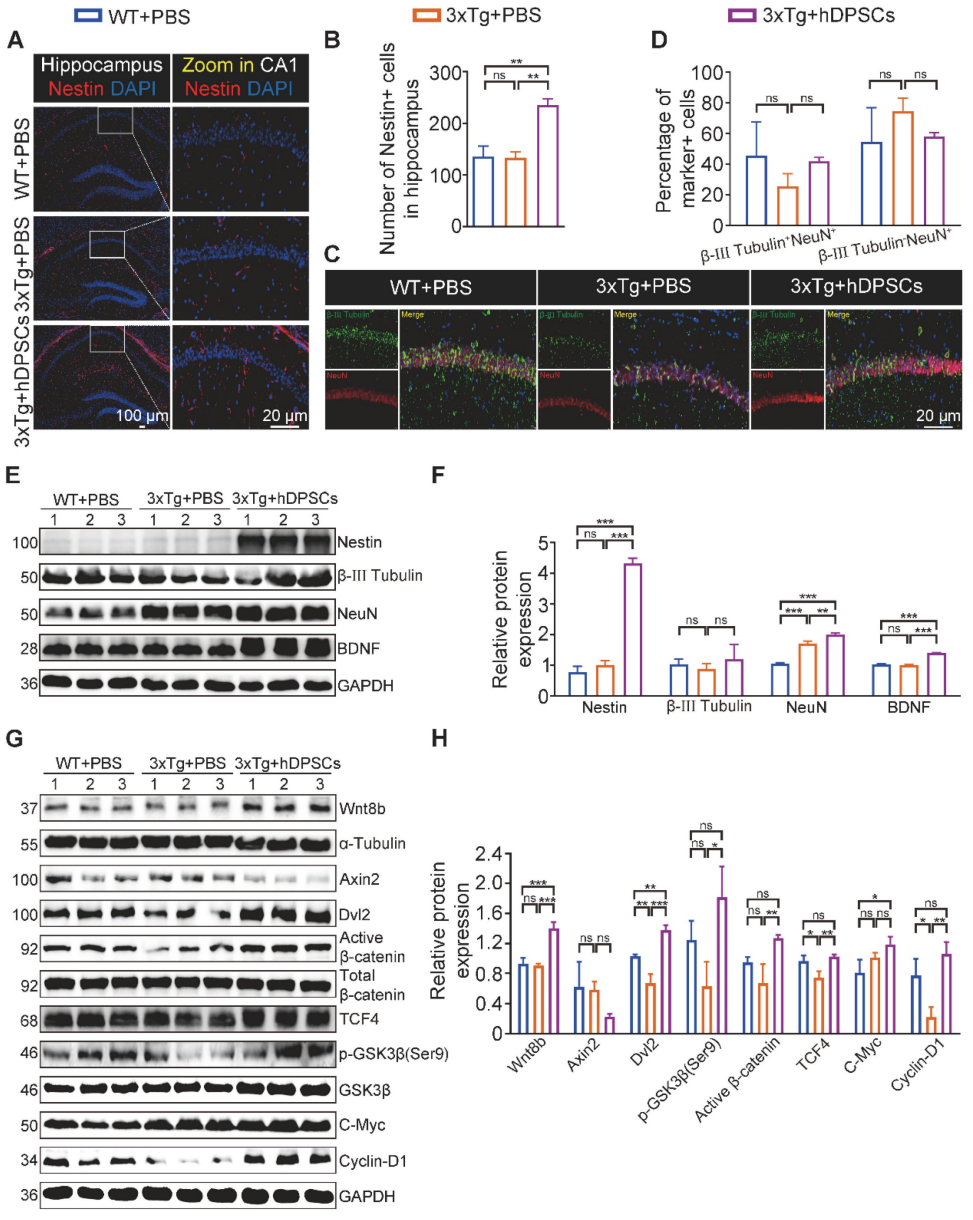
** hDPSCs partially imparted sustained hippocampal neural regeneration in 3xTg-AD mice over time.** (A, B) Representative immunostaining and quantification of Nestin (red)-positive cells in brain sections of the hippocampus from mice 6 months after therapy. Scale bar = 100 µm, 20 µm. (C) Representative co-immunostaining of β-III Tubulin (green) and NeuN (red) in brain sections of the hippocampus (CA1) from mice 6 months after therapy. Scale bar = 20 µm. (D) Shown in quantification is a summary of the distribution of neurons at different maturation stages. (E, F) The protein expression levels and quantification of Nestin, β-III Tubulin, NeuN and BDNF in the hippocampus from mice 6 months after therapy were tested by western blotting. (G) Western blotting results showed the protein expression levels of canonical Wnt pathway targets in the hippocampus from mice 6 months after therapy. (H) Quantitative Western blotting was performed for the relative protein expression of canonical Wnt pathway targets in the hippocampus from mice 6 months after therapy. (n = 3 mice per group; all data shown as mean ± SD, **P < 0.01, ***P < 0.001).

To further explore whether the prolonged cognitive improvement was still accompanied by sustained neural regeneration, we then examined key marker proteins involved in neurons in the hippocampus of mice by immunofluorescence. We found an outstanding increase in the number of NSC marker Nestin-positive cells in the hippocampus of hDPSCs-treated mice, even higher than that in WT+PBS littermates (Figure 8A, B).

Massive recruitment and activation of NSCs have an important role for hippocampal neurogenesis and repair of the degenerated or injured brain 35. We then sought to identify whether the differential expression of NSCs led to a net change in the number of mature neurons. Quantitative analysis showed a nearly two-fold increase in the percentage of β-III Tubulin^+^NeuN^+^-labeled neurons among the WT+PBS and hDPSCs-treated mice compared to AD+PBS mice, with no statistically significant difference (Figure 8C, D). But a significant increase in the number of β-III Tubulin^+^NeuN^+^-labeled neurons was observed in the DG region of hDPSC-treated mice compared with AD+PBS mice (Figure S19A, B). This may be closely related to the expression of active NSCs in this region. Subsequently, the protein expression of nerve cells in the hippocampus was further verified by western blotting (Figure 8E, F). The results indicated that the protein expression of Nestin was obviously higher than that of WT+PBS and AD+PBS mice, which was consistent with the fluorescence results. The protein expression of β-III Tubulin, NeuN, and BDNF for hDPSCs-treated mice was only slightly increased relative to that of AD+PBS mice. This prolonged effect on NSCs activation might underlie hDPSCs-induced sustained hippocampal neural regeneration in 3xTg-AD mice, which is absent from AD+PBS mice.

Considering that transplanted hDPSCs can spontaneously differentiate into neurons within a short term and that secretion of Wnt8b activates the canonical Wnt signaling pathway to promote hippocampus neural regeneration, we wondered whether human-derived neurons and cells could survive in 3xTg-AD mice for a long term with similar Wnt signaling activation in the hippocampus. In sharp contrast to the short-term graft outcomes, few double-labeled Stem121^+^NeuN^+^ neurons were detectable in the hippocampus, while Stem121^+^IBA1^+^ microglial cells and stem121^+^GFAP^+^ astrocytes were present in little abundance after 6 months of administration (Figure S20), suggesting that hDPSCs-derived neurons and cells appear to be refractory to long-term survival under persistent pathological insults of AD. Nevertheless, we found hDPSCs still enhanced Wnt8b and its downstream receptors expression in hippocampal tissue and that the differential expression of most genes was statistically significant, including the target genes β-catenin, TCF4, and Cyclin-D1, which are associated with proliferation and neural regeneration (Figure 8G, H). Together, these studies suggested that a single intracerebral administration with hDPSCs partially mediated sustained hippocampal neural regeneration over a long period of time, with potential and specific roles for activation of the canonical wnt signaling pathway.

## Discussion

To date, considerable attention has been toward the feasibility of stem cell therapy for treating AD by eliminating the destructive impacts of toxic substances in the brain 9. Substantial studies has provided strong evidence that transplanted stem cells, particularly MSCs, mediate immune and inflammatory responses in the brain microenvironment through innate immunomodulatory properties or the secretion of neurotrophic/neuroprotective cytokines, while differentiating into neuronal cells to replace damaged neurons, thereby alleviating AD neuropathological symptoms and stimulating endogenous neuronal repair 15, 35, 36. The multifunctionality of stem cell therapies for the simultaneous management of multiple AD pathologies may be a glimmer of hope for addressing the repeated failures of current anti-AD drugs therapeutics. Currently, a number of clinical trials involving hNSCs, hBM-MSCs, and hUC-MSCs registered with ClinicalTrials.gov for the treatment of AD are underway 9.

To ensure the efficacy and application prospects of stem cells in AD patients, it is critical to evaluate relevant factors such as cell accessibility, stability, proliferative properties, and cryopreservation. Human NSCs with optimal capacity to differentiate into different neural cells are produced only in the subventricular zone and sub-granular zone of the hippocampal formation, which is difficult to accessible 37. The readily available MSCs and their possible therapeutic potential are therefore of particular interest. Attention has been drawn to the fact that hDPSCs migrated from cranial neural crest lineage can be easily isolated from impacted third molars or orthodontic teeth 9. The first passage of hDPSCs showed extraordinary proliferative capacity and survival rate 38. More importantly, hDPSCs not only have a trilineage differentiation capacity similar to that of MSCs from other sources like BM-MSCs and AD-MSCs but also show fantastic neurogenic differentiation potential 39. hDPSCs not only highly express neural-related biomarkers such as nestin, but further differentiate into functionally active neuronal cells, dopaminergic-like cells, and oligodendrocyte-like cells under the neural microenvironment 9. It is worth mentioning that hDPSCs still maintain their biological properties after long-term cryopreservation and allow direct storage of whole teeth in a GMP-grade teeth bank for emergencies. The quality of clinical-grade hDPSCs certified by national authority used in this study also greatly guarantees the reliability of these research data and provides a structural basis for further clinical research.

Given that AD has a significant preclinical phase of at least 10-20 years prior to onset of clinical symptoms 4 and that recent clinical studies have shown that early transplantation of MSCs has obvious benefits for newly diagnosed type-1 diabetes patients compared to late transplantation in patients 40. Simultaneously, targeted treatments for primary Aβ species rather than advanced plaques have also shown success in preventing the onset of AD symptoms 41. We sought to investigate the impact of early intervention on AD progression by transplanting hDPSCs at the early stage of disease progression in 3xTg-AD mice 42. In the present study, we demonstrated that early single intracranial administration of clinical-grade human-derived DPSCs fully restored memory in 3xTg-AD mice and produced impressive neuropathological changes at multiple critical levels. Meanwhile, after a single treatment, the AD mice still maintained better cognitive function in the long-term disease stage, with partial sustained improvement of disease features. Extensive studies have shown that hyperphosphorylated tau and NFTs seem to be closely associated with neuronal loss and cognitive deficits 43 and that targeted therapy of tau pathology seems to be more effective than single Aβ therapy 44. An emerging report also suggests that a tau-targeting nano chaperone can effectively improve cognitive deficits and pathology in OA-induced AD mice 45. Therefore, in the treatment exploration of the AD model *in vitro*, we established cell co-culture models of hDPSCs with OA-induced nerve cells and SH-SY5Y cells transfected with plasmids expressing the same mutant forms of APP and Tau as those in 3xTg-AD mice. In these models, we demonstrated that hDPSCs could effectively alleviate the typical pathological characteristics of AD and rescue neurological damage, including mitochondrial structure and function.

The neuropathology has encroached upon the entorhinal cortex and hippocampus in the early stages of AD progression, destructing adult neurogenesis and thus hippocampal modulation of learning and memory 46. Functional magnetic resonance imaging (fMRI) studies of AD progression *in vivo* and AD postmortem studies have shown that neuronal damage in the CA1 region and the subiculum are the most affected regions in the hippocampus 22. While current pharmacological therapies are helpless against neuronal damage in the brain, another promising finding of stem cell therapy for AD is effective replacement mechanisms and enhanced neurogenesis in the hippocampus. hDPSCs derived from the embryonic neural crest exhibit excellent neural differentiation and neuroprotection potential, second only to embryonic stem cells. Just as we found in this study, the beneficial effects of hDPSCs on cognition were not only reflected in mediating the alteration of Aβ, tau pathology, and brain inflammation but also in eliciting robust endogenous NSCs reactivation and accelerated maturation of new neurons in the CA1 and DG regions, thus restoring hippocampal-dependent cognition. Importantly, a great number of functionally mature human neurons (GABAergic neurons and cholinergic neurons) were observed around the injection site in the hippocampus of hDPSCs-treated mice, suggesting that hDPSCs implanted into the mouse brain have the ability to efficiently differentiate into neurons and possibly replace damaged neurons 47. AD mice injected with hDPSCs also exhibited higher levels of BDNF and increased density of hippocampal dendritic spines. This result ties well with previous studies wherein NSC transplantation mediated improved cognitive function in 3xTg-AD mice by elevating BDNF expression in the hippocampus 48.

What are the potential pathways mediating the ability of endogenous neural regeneration in the hippocampus? We performed RNA-sequencing analysis of adult mouse hippocampus with or without hDPSCs treatment to analyze and search for possible mediators. We found that the expression of Wnt8b, a gene critical to the development and differentiation of forebrain structure 31, was significantly increased in the hippocampus. Wnt signaling activity regulates diverse developmental processes in the embryonic brain and controls the homeostasis of the hippocampal niche, including the proliferation and differentiation of progenitor cells and the neurogenetic potential 49. Interestingly, retention of the canonical Wnt signaling cascade in adult dentate gyrus is effectively inhibited by AD pathology 24, 50, and hDPSCs may be an specifically exogenous activator that triggers the Wnt8b/β-catenin signaling cascades to stimulate NSC proliferation and neurogenesis. Furthermore, the conduction of the canonical Wnt signaling continues a considerable period of time. The recombinant protein Wnt8b was also able to activate the Wnt/β-catenin signaling cascades in mouse primary neurons *in vitro* and accelerate the maturation process of primary neurons, directly corroborating the *in vivo* studies.

Although we have revealed that Wnt8b is related to the efficacy of hDPSCs in promoting adult hippocampal neural regeneration, we believe that Wnt8b may serve as a cue or clue for the treatment of AD. However, we also believe that there might be other bioactive factors besides Wnt8b involving in the systemic and integrated effects of hDPSCs. Since we used neural crest-derived MSCs, we focused on exploring the effects of hDPSCs on adult hippocampal neural regeneration, while the regulatory mechanisms of AD pathology still require further investigation. Our histological analysis of neural regeneration in the mouse hippocampus was not systematic, and further powered single-cell transcriptomic analysis and electrophysiological studies will be required to systematically explore the lineage tracing of hDPSCs in the adult hippocampal niche and the integration of differentiated cells into existing neural network activities. Furthermore, intracranial transplantation also inevitably causes mechanical injury, and the limited number of stem cells delivered makes multiple administrations of intracerebral transplantation less feasible for clinical transformation. Finding the optimal route of administration to better adapt to clinical applications is also the focus of our next research.

In conclusion, we proved for the first time that the early administration with clinical-grade hDPSCs can improve neuropathological features and adult hippocampal neural regeneration to restore cognitive deficits in 3xTg-AD mice. The long-term therapeutic efficacy of hDPSCs after a single transplantation was also impressive, despite rampant pathology. Our study not only provides evidence supporting the advantages of stem cell-based therapies but also links oral health to systemic disease, suggesting that the development of oral-derived hDPSCs can provide clues and cues for preventing the increasing prevalence of AD.

## Supplementary Material

Supplementary figures and tables.
