# Supplementary material for: Clinical-grade human dental pulp stem cells improve adult hippocampal neural regeneration and cognitive deficits in Alzheimer's disease
Source: Theranostics. 2025 Jan 1;15(3):894–914. doi: 10.7150/thno.102315 (PMC11700856; doi:10.7150/thno.102315)
Supplement: Supplementary file 1 — Supplementary figures and tables. [file thnov15p0894s1.pdf]

Supporting information

Clinical-grade human dental pulp stem cells improve adult hippocampal neural regeneration and cognitive deficits in Alzheimer's Disease

Wei Xiong[1,5, †], Wenting She[1,†], Ye Liu[1,†], Heng Zhou[1,†], Xinxin Wang[2], Fang Li[3], Ruohan Li[1], Junnan Wang[1], Dongdong Qin[3], Shuili Jing[1], Xingxiang Duan[1], Cailei Jiang[1], Chun Xu[4], Yan He[2, *], Zhihao Wang[3,**], Qingsong Ye[1,4,*]**

Supplementary Figures

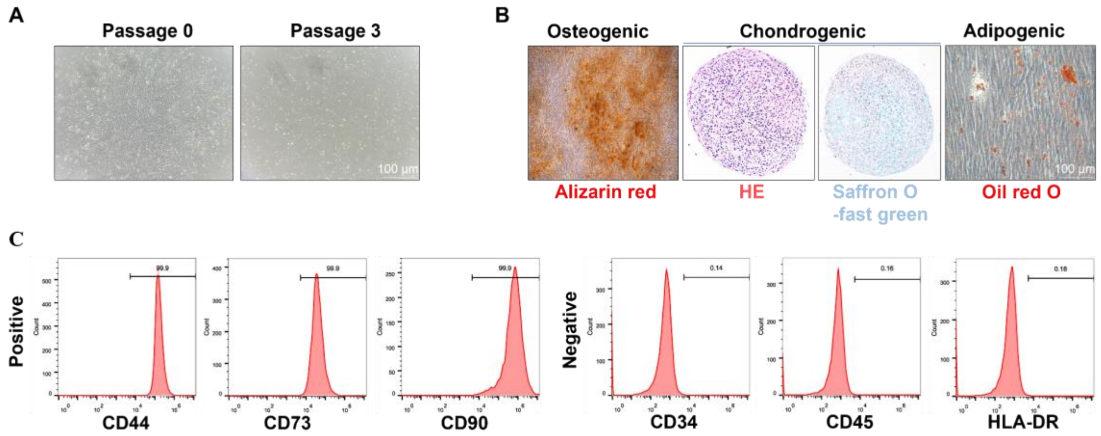

Figure S1. The morphology, phenotype, and differentiation potential of clinical-grade hDPSCs were characterized. (A) The morphology of hDPSCs (P0 or P3) were observed under light microscopy. Scale bar=100 μm. (B) The multi-lineage differentiation potential of hDPSCs were tested. Scale bar=100 μm. (C) Flow cytometry showed hDPSCs were positive for mesenchymal lineage markers (CD44, CD73 and CD90), negative for hematopoietic markers (CD34, CD45 and HLA-DR).

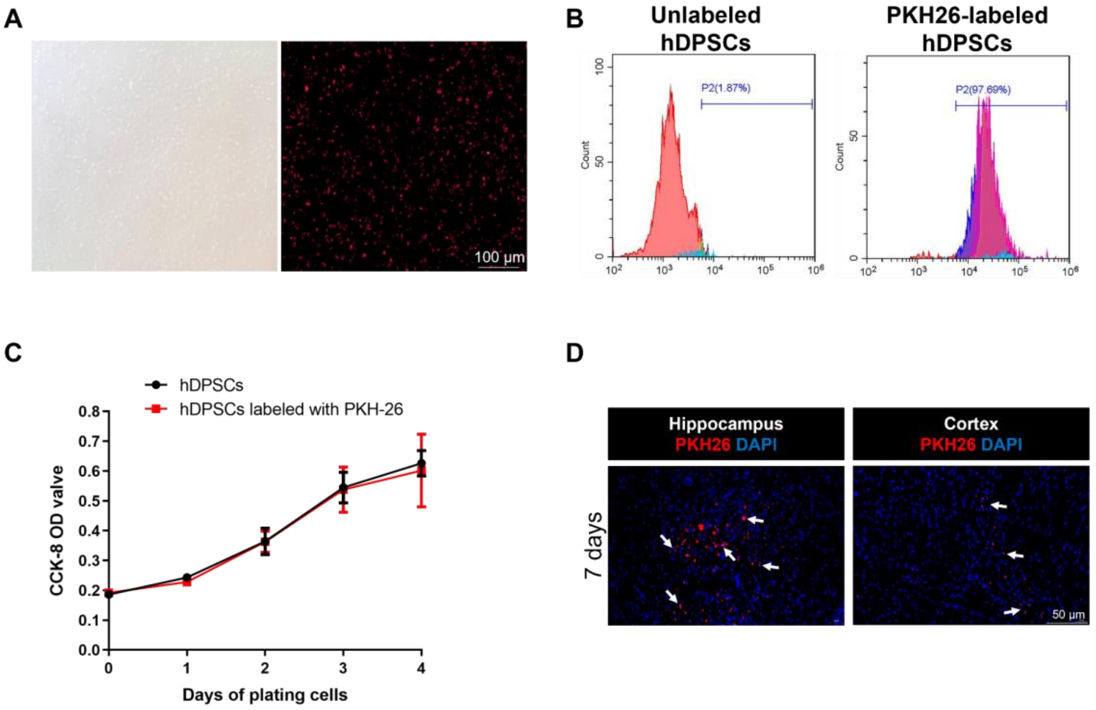

Figure S2. The staining effectiveness and toxicity of PKH26-labeled hDPSCs in vitro and the accuracy of transplantation in the brain of 3xTg-AD mice were evaluated. (A) PKH26-labeled hDPSCs and unlabeled DPSCs were observed under light microscopy. Scale bar=100 μm. (B) Analysis of staining effectiveness by flow cytometry. (C) The proliferative comparison by Cell Count-8 Kits (CCK-8) between the PKH26-labeled hDPSCs and unlabeled hDPSCs. (D) PKH26-labeled hDPSCs (red, white arrows) and DAPI (blue) immunostaining showed the location of hDPSCs in the hippocampus and cortex of 3xTg AD mice. Scale bar=50 μm. (n=3 per group; Values represented mean ± SD).

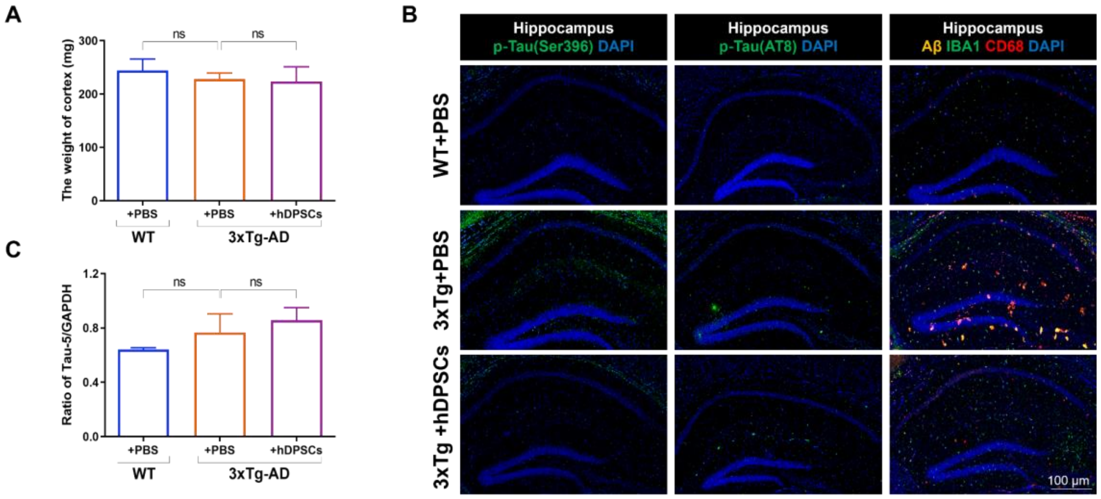

Figure S3. hDPSCs ameliorated neuropathological features in the hippocampus of 3xTg-AD mice. (A) At the end of the behavioral experiment of the 5-week treatment cycle, the cortex of partial mice was isolated and weighed. (B) Representative immunostaining of p-Tau (Ser396 and AT8, green) and Aβ (orange), IBA1 (green), and CD68 (red) in the hippocampus from mice 5 weeks after therapy. Scale bar=100 μm. (C) Quantification comparison of protein expression of Tau-5 compared with GAPDH in the hippocampus. (n=3 mice per group; Values represented mean ± SD).

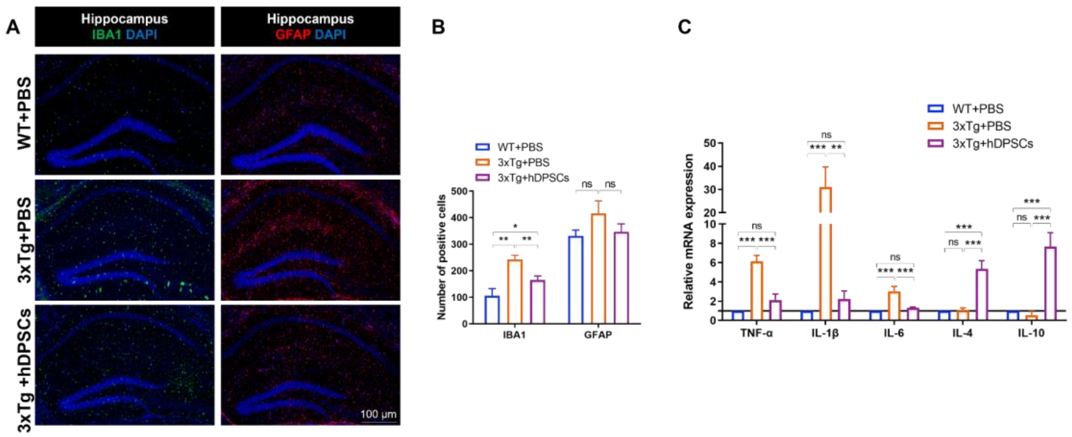

Figure S4. hDPSCs ameliorated neuropathological features in the hippocampus of 3xTg-AD mice. (A, B) Representative immunostaining and quantification of IBA1 (green) and GFAP (red)-positive cells in brain sections of the hippocampus from mice 5 weeks after therapy. Scale bar=100 µm. (C) Quantitative real-time PCR analysis was performed for the relative mRNA expression of inflammation factor genes in the hippocampus from mice 5 weeks after therapy. (n=3 mice per group; Values represented mean ± SD, *P < 0.01, **P < 0.01, ***P < 0.001).

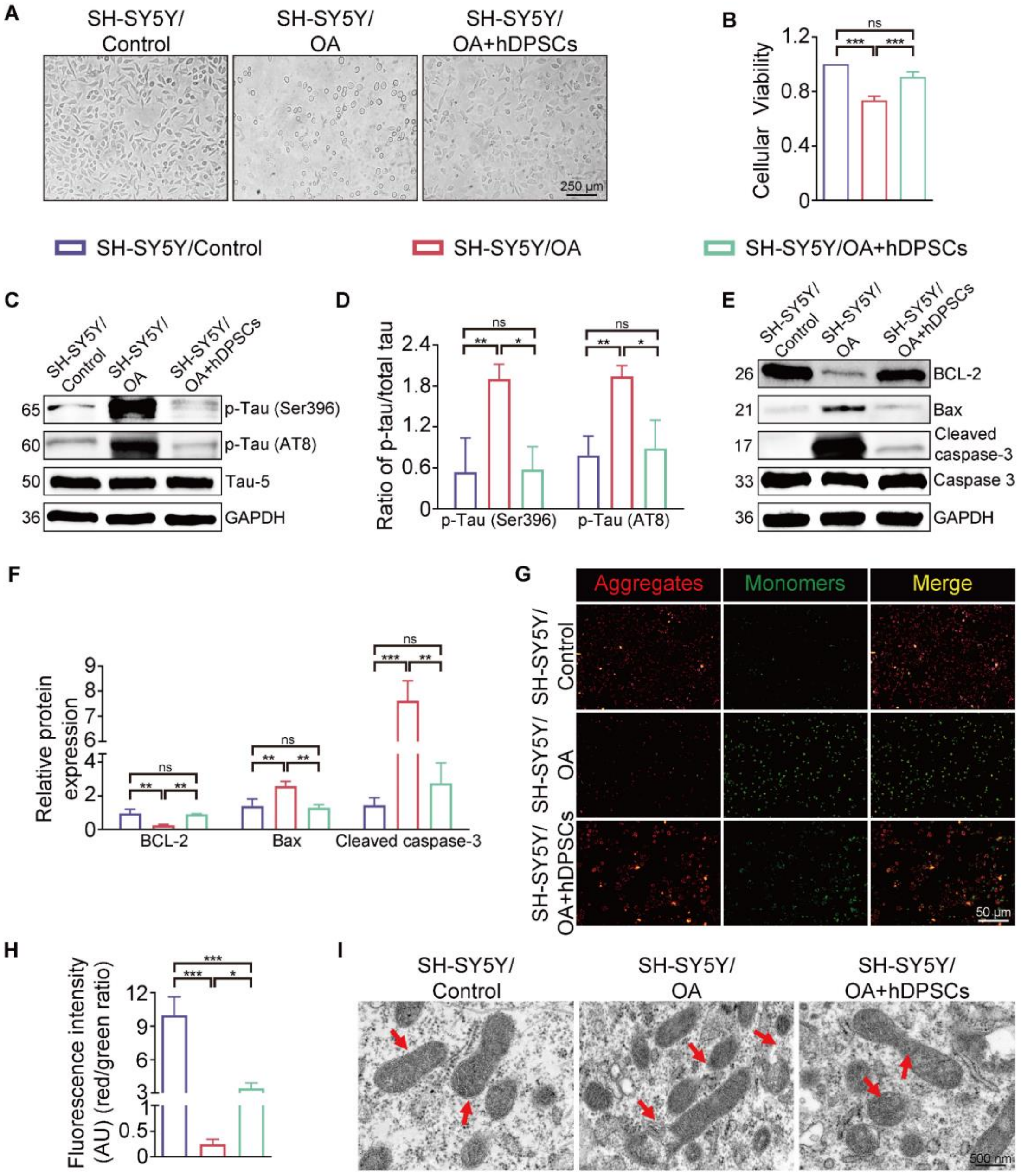

Figure S5. hDPSCs effectively alleviated okadaic acid-induced SH-SY5Y cells damage in vitro. (A) Representative morphological images of SH-SY5Y cells in the three groups were observed under light microscopy. Scale bar =250 μm. (B) The cell viability of AD model SH-SY5Y cells was determined using a CCK-8 assay. (C, D) The protein expression levels and quantification of phospho-Tau (Ser396 and AT8) in the SH-SY5Y cells of the AD model were tested by western blotting. (E, F) The expression levels and quantification of apoptosis-associated proteins BCL2, Bax, caspase3, and cleaved caspase-3 in the SH-SY5Y cells of the AD model were tested by

western blotting. (G, H) Mitochondrial membrane potential of the SH-SY5Y cells were detected by the JC-1 staining and the quantification of red/green fluorescence intensity. Scale bar =50 μm. (I) Morphometric ultrastructural analyses by transmission electron microscopy (TEM) showed the intracellular mitochondrial structure of SH-SY5Y in the three groups. Scale bar =500 nm. (n=3 per group; Values represented mean ± SD, *P < 0.05, **P < 0.01, ***P < 0.001).

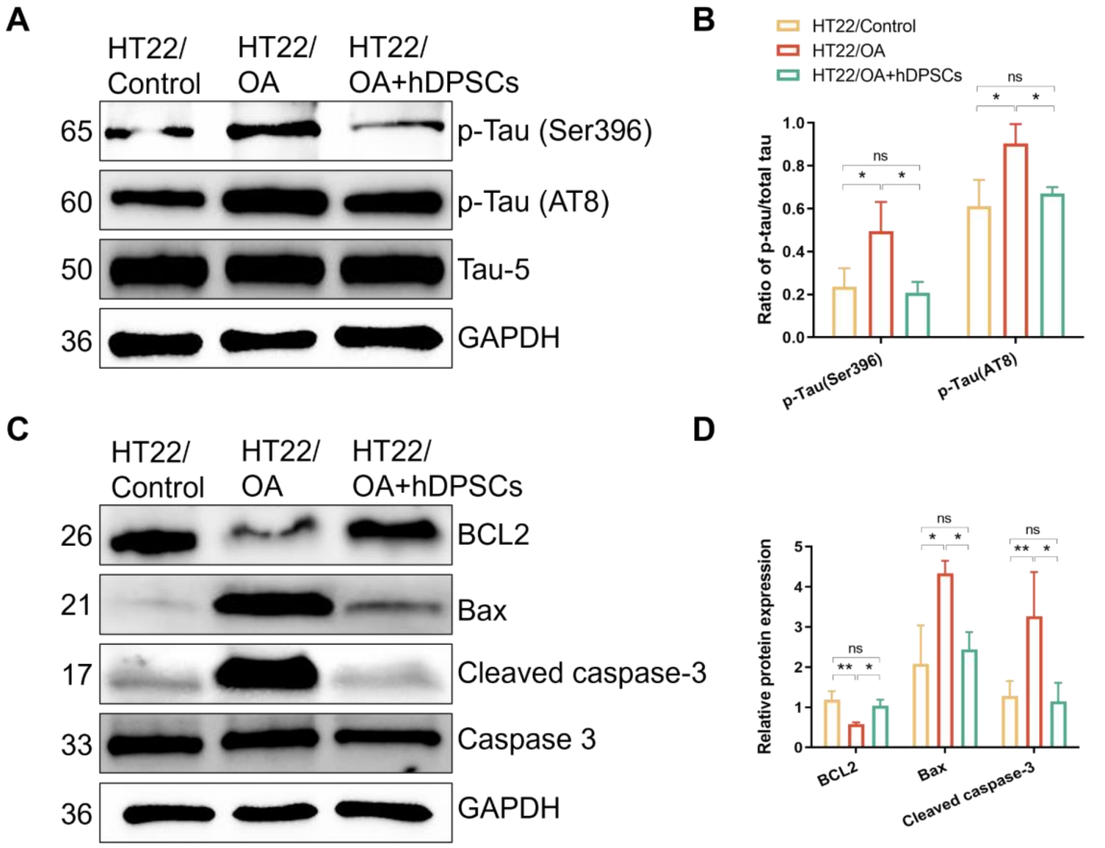

Figure S6. hDPSCs effectively alleviated okadaic acid-induced HT22 cells damage in vitro. (A, B) The protein expression levels and quantification of phospho-Tau (Ser396 and AT8) in the HT22 cells of the AD model were tested by western blotting. (C, D) The expression levels and quantification of apoptosis-associated proteins BCL2, Bax, caspase3, and cleaved caspase-3 in the HT22 cells of the AD model were tested by western blotting. (n=3 per group; Values represented mean ± SD, *P < 0.05, **P < 0.01).

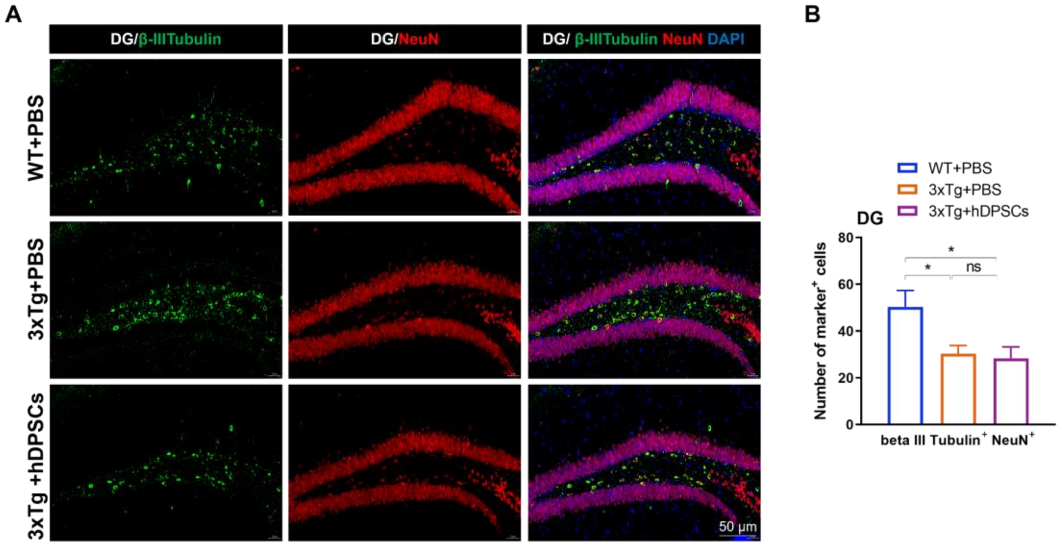

Figure S7. hDPSCs rejuvenated endogenous neural regeneration in the hippocampus of 3xTg-AD mice. (A) Representative co-immunostaining of β-III Tubulin (green) and NeuN (red) in the dentate gyrus (DG) of the hippocampus from mice 5 weeks after therapy. (B) Shown in quantification is a summary of the distribution of neurons at different maturation stages. Scale bar=50 μm. (n=3 mice per group; Values represented mean ± SD, *P < 0.05).

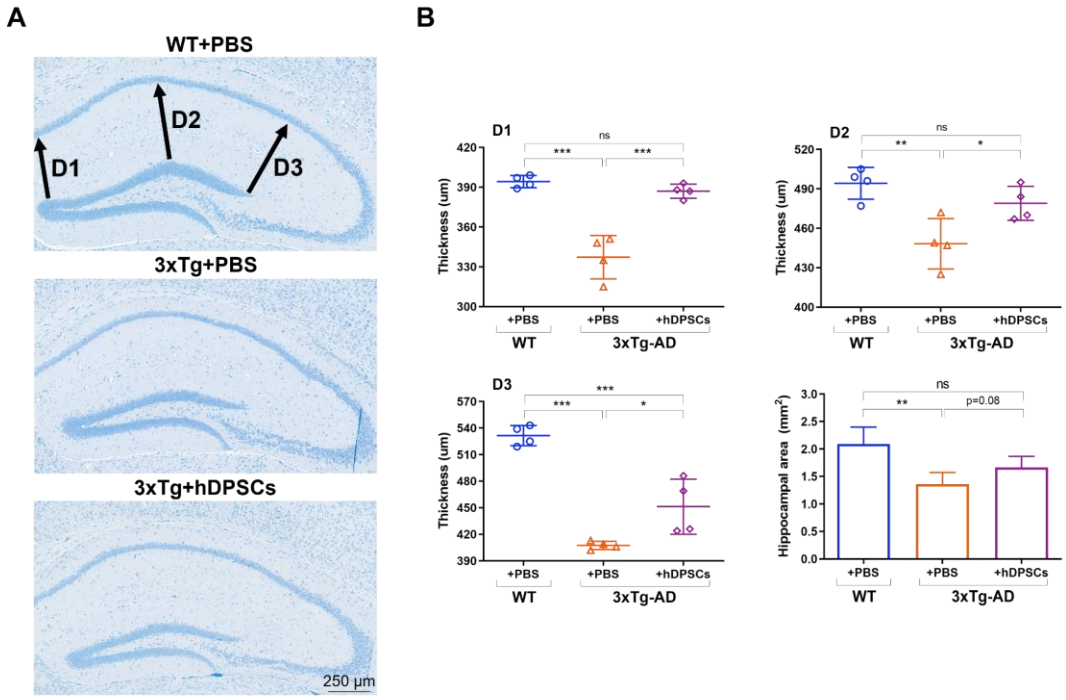

Figure S8. hDPSCs alleviated hippocampal atrophy in 3xTg-AD mice. (A) Images of Nissl-stained brain slices in which headed arrows at D1, D2 and D3 indicated where thickness was measured between the CA1 and dentate gyrus (DG) hippocampal sub-regions. Scale bar=250 μm. (B) Quantification of neuropil thickness for the D1/2/3 selected and determination of hippocampal area. (n=3 mice per group; Values represented mean ± SD, *P < 0.05, **P < 0.01, ***P < 0.001).

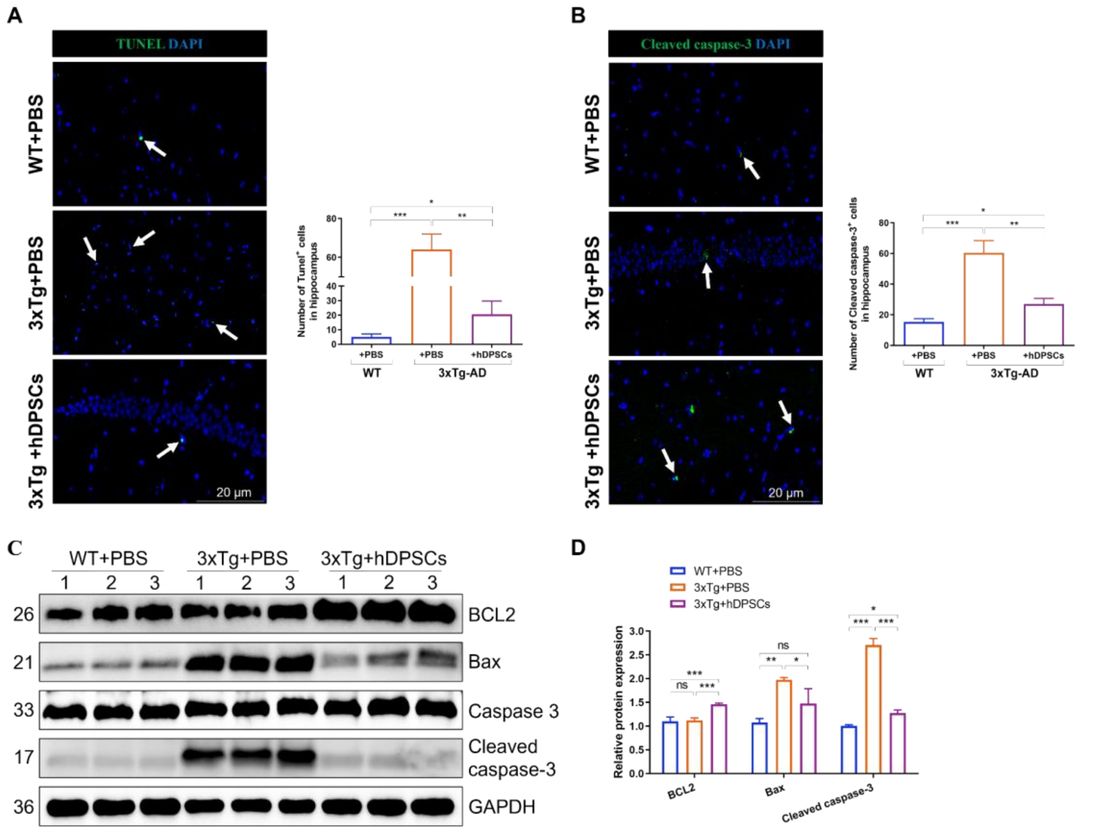

Figure S9. hDPSCs reduced the apoptosis of hippocampal cells in 3xTg-AD mice 5 weeks after therapy. (A) The Tunel staining and quantification of positive cells in the hippocampus from mice 5 weeks after therapy. Scale bar=20 μm. (B) Representative immunostaining and quantification of cleaved caspase-3 (green)-positive cells in brain sections of the hippocampus from mice 5 weeks after therapy. Scale bar=20 μm. (C, D) The expression levels and quantification of apoptosis-associated proteins BCL2, Bax, caspase3, and cleaved caspase-3 in the hippocampus from mice 5 weeks after therapy were tested by western blotting. (n=3 mice per group; Values represented mean ± SD, *P < 0.05, **P < 0.01, ***P < 0.001).

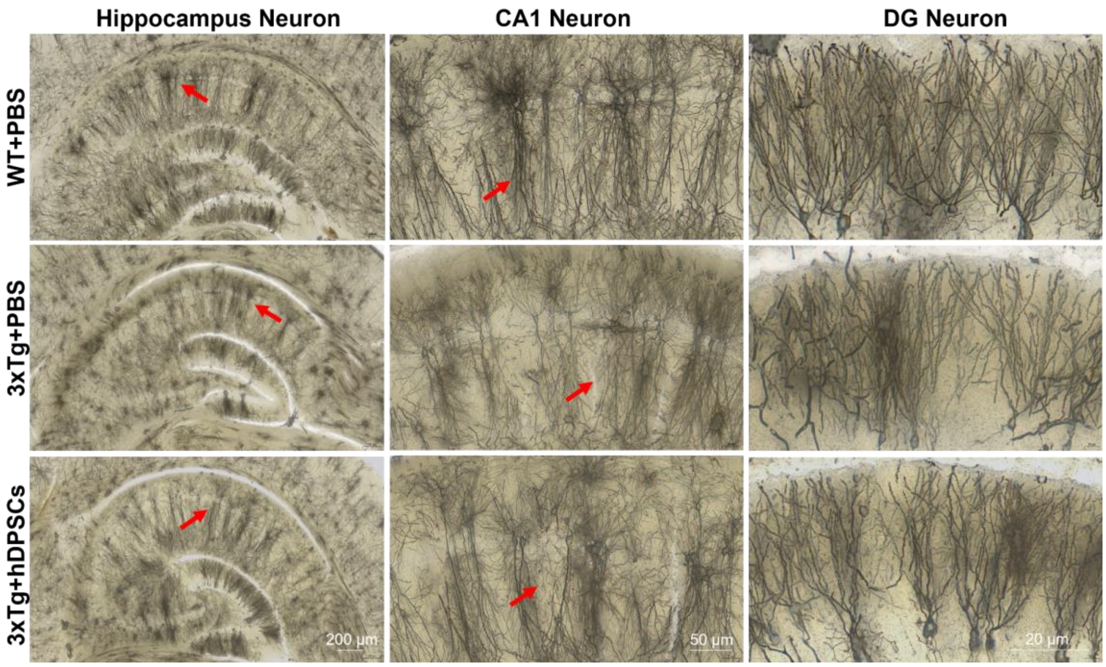

Figure S10. hDPSCs rejuvenated endogenous neural regeneration in the hippocampus of 3xTg-AD mice. Representative Golgi staining of spine density in the CA1 and DG of the hippocampus from mice 5 weeks after therapy. Scale bar=200 μm, 50 μm, 20 μm.

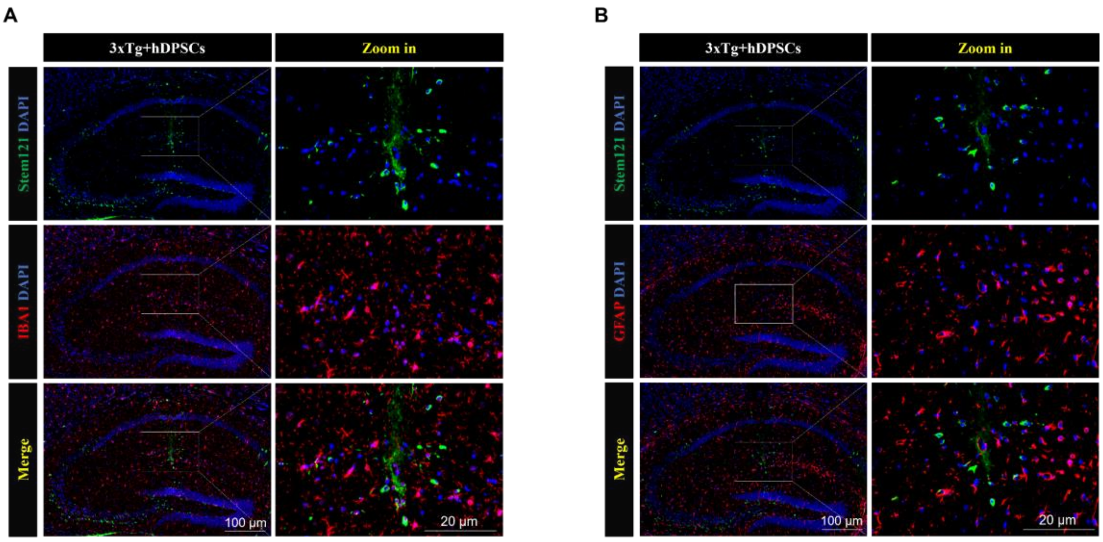

Figure S11. Specific human cell markers were detected in the hippocampus from mice 5 weeks after treatment. (A, B) Representative co-immunostaining of Stem121 (green) and IBA1(red)/GFAP (red) to detect the differentiation status of hDPSCs in brain sections of the hippocampus from mice 5 weeks after therapy. Scale bar=100 µm, 20 µm.

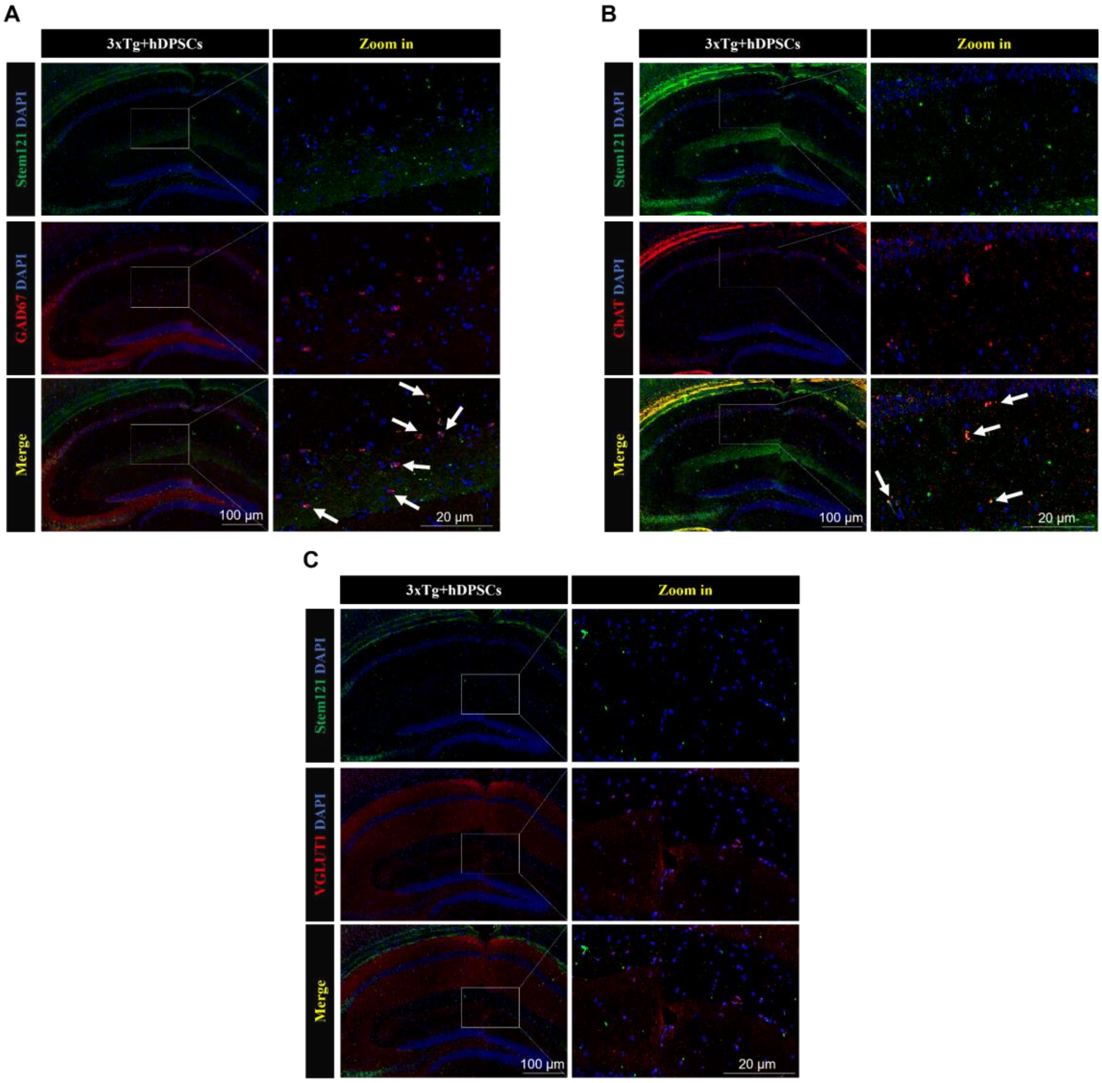

Figure S12. Neural differentiation of transplanted hDPSCs in the hippocampus from mice 5 weeks after treatment. (A, B, C) Immunofluorescence analysis of subtypes of hDPSCs-derived neurons among Stem121$^+$ cells in brain sections of the hippocampus from mice 5 weeks after therapy. Scale bar=100 μm, 20 μm.

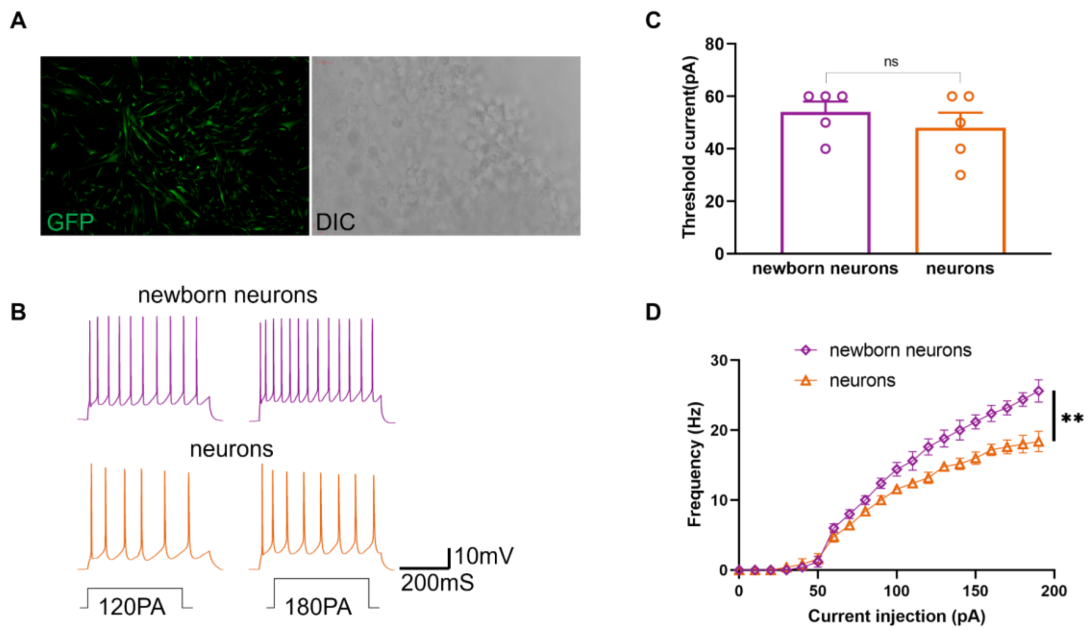

Figure S13. The functional changes of transplanted hDPSCs-derived neurons in hippocampus. (A) GFP-hDPSCs and Representative hDPSCs-derived neurons in hippocampus on acute slice from the brain of 3xTg-AD mouse 5 weeks after treatment. (B, C) APs generation in transplanted hDPSCs-derived neuronal cells and original neuronal cells in responses to step current injection. (D) Comparison of Force-Intensity curves from transplanted hDPSCs-derived neuronal cells and original neuronal cells. (n=5 per group; Values represented mean ± SD, $**P < 0.01$).

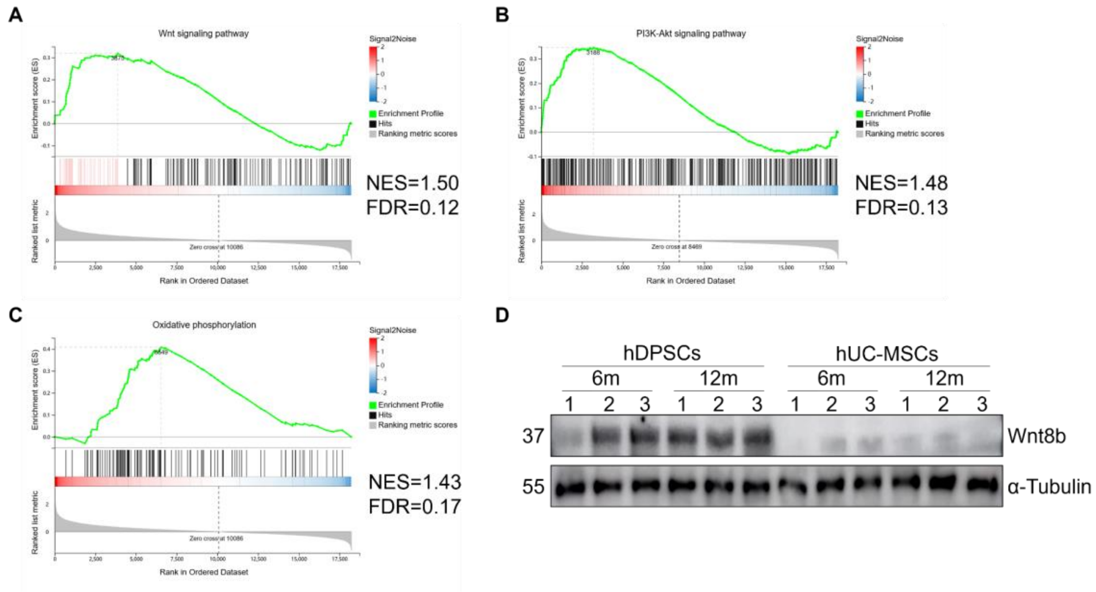

Figure S14. hDPSCs regulating adult hippocampal neural regeneration via activation of canonical Wnt/β-Catenin Pathway. (A, B, C) Gene Set Enrichment Analysis (GSEA) of differentially expressed genes (DEGs) between hDPSCs treatment group and PBS control group in 3xTg AD mice. (D) Western blotting results showed the protein expression levels of Wnt8b in hDPSCs and hUC-MSCs stimulated with brain homogenate from 6- and 12-month mice. (n=3 per group).

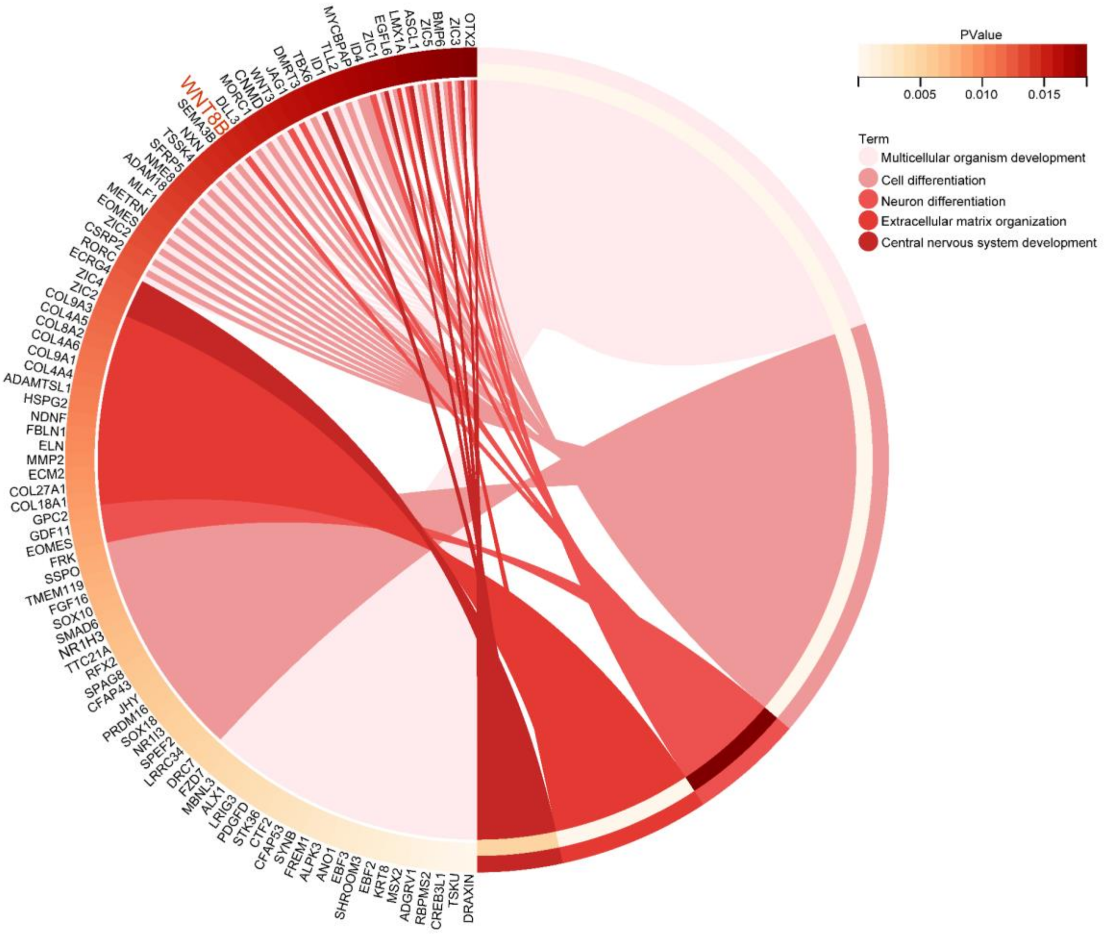

Figure S15. hDPSCs regulating adult hippocampal neural regeneration via activation of canonical Wnt/β-Catenin Pathway. Significant enriched Gene Ontology (GO) terms of differentially expressed genes (DEGs) between hDPSCs treatment group and PBS control group in 3xTg-AD mice based on their functions.

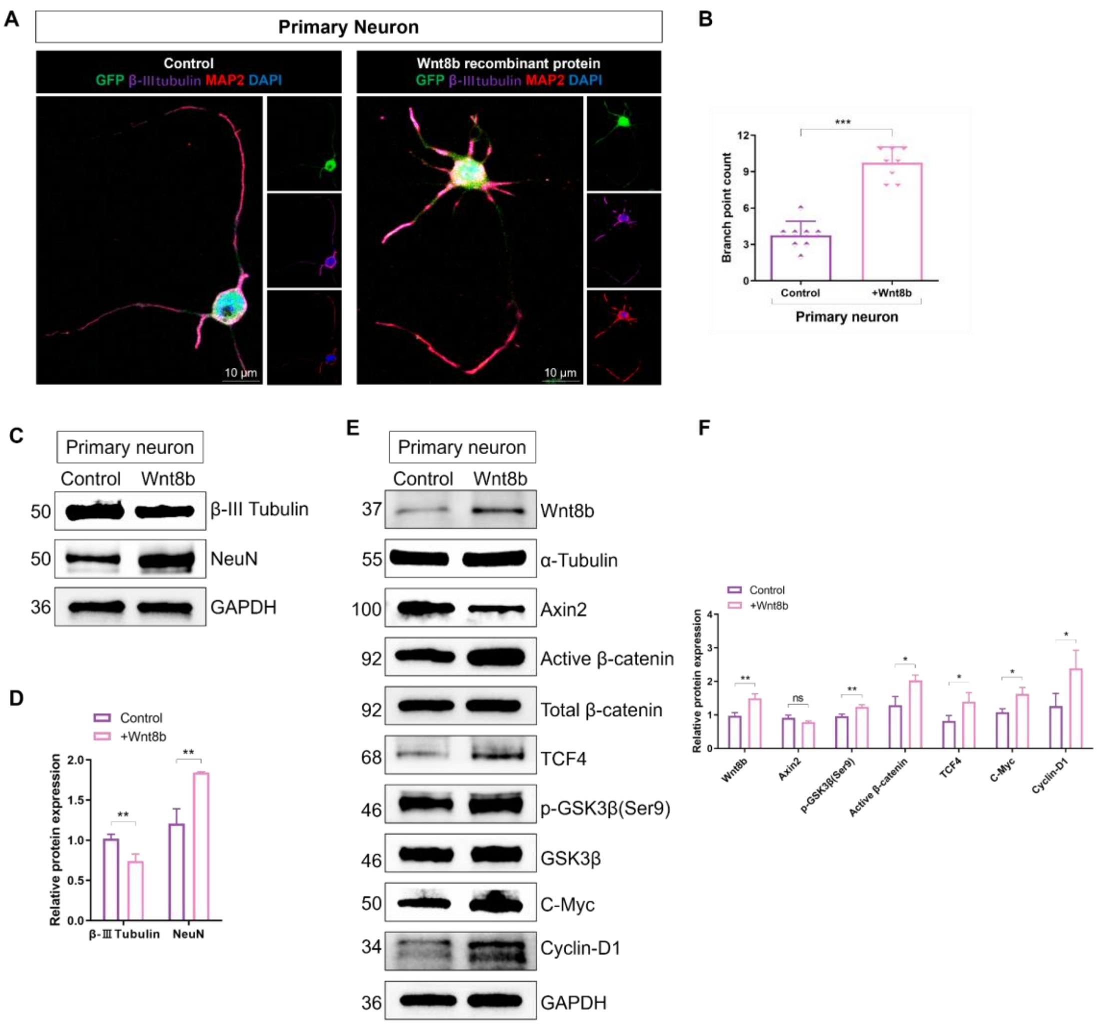

Figure S16. Wnt8b accelerated the maturation of primary mouse neurons. (A, B) Representative co-immunostaining (GFP, green; β-III Tubulin, purple; and MAP2, red) and quantification of branches in primary mouse neurons after wnt8b therapy. (C, D) Western blotting results showed the protein expression levels and quantification of β-III Tubulin and NeuN in the primary mouse neurons after wnt8b therapy. (E, F) The protein expression levels and quantification of canonical Wnt pathway targets in the primary mouse neurons after wnt8b therapy were tested by western blotting. Scale bar=10 μm. (n=3-8 per group; Values represented mean ± SD, *P < 0.05, **P < 0.01, *** P < 0.001).

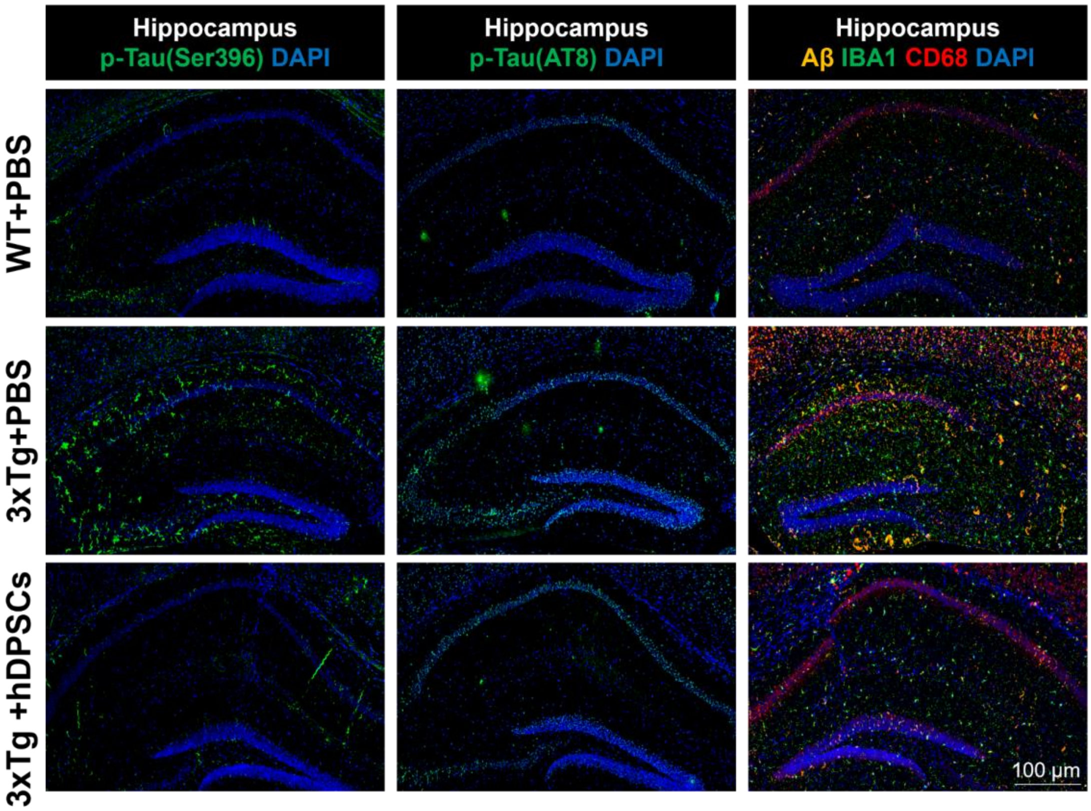

Figure S17. A single intracerebral treatment with hDPSCs attenuated neuropathological progression in 3xTg-AD mice in the long term. Representative immunostaining of p-Tau (Ser396 and AT8, green) and Aβ (orange), IBA1 (green), and CD68 (red) in the hippocampus from mice 6 months after therapy. Scale bar=100 μm.

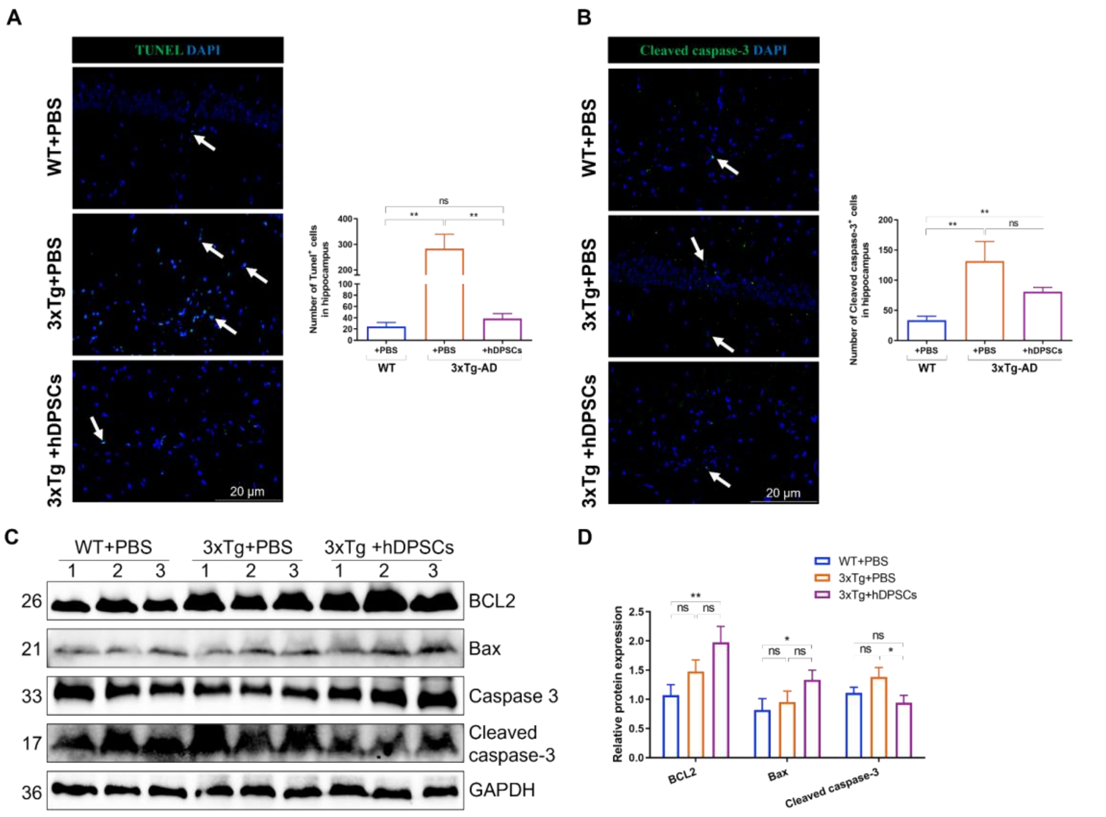

Figure S18. hDPSCs partially reduced the apoptosis of hippocampal cells in 3xTg-AD mice 6 months after therapy. (A) The Tunel staining and quantification of positive cells in the hippocampus from mice 6 months after therapy. Scale bar=20 μm. (B) Representative immunostaining and quantification of cleaved caspase-3 (green)-positive cells in brain sections of the hippocampus from mice 6 months after therapy. Scale bar=20 μm. (C, D) The expression levels and quantification of apoptosis-associated proteins BCL2, Bax, caspase3, and cleaved caspase-3 in the hippocampus from mice 6 months after therapy were tested by western blotting. (n=3 mice per group; Values represented mean ± SD, *P < 0.05, **P < 0.01).

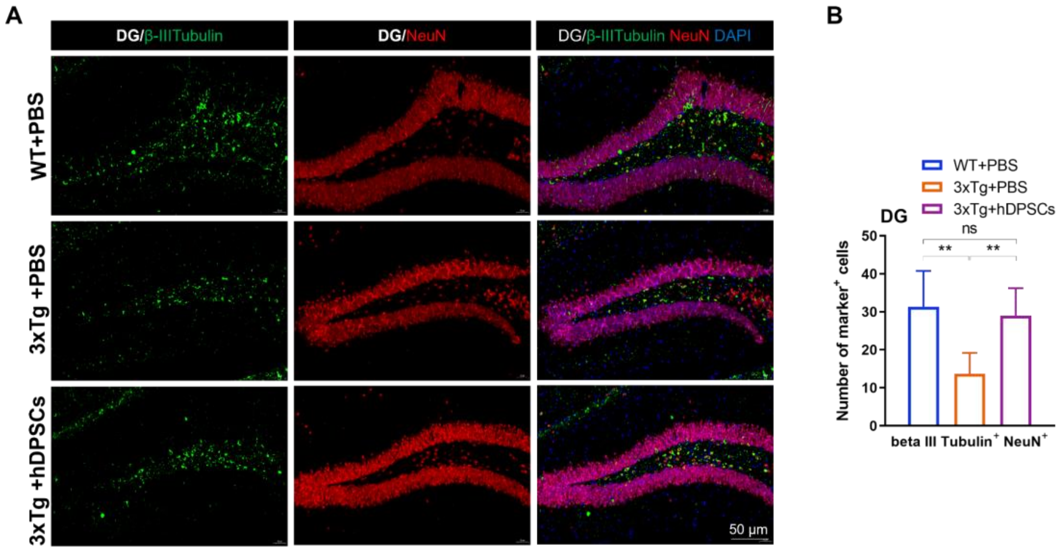

Figure S19. hDPSCs partially imparted sustained hippocampal neural regeneration in 3xTg-AD mice over time. (A) Representative co-immunostaining of β-III Tubulin (green) and NeuN (red) in the dentate gyrus (DG) of the hippocampus from mice 6 months after therapy. (B) Shown in quantification is a summary of the distribution of neurons at different maturation stages. Scale bar=50 μm. (n=3 mice per group; Values represented mean ± SD, **P < 0.01).

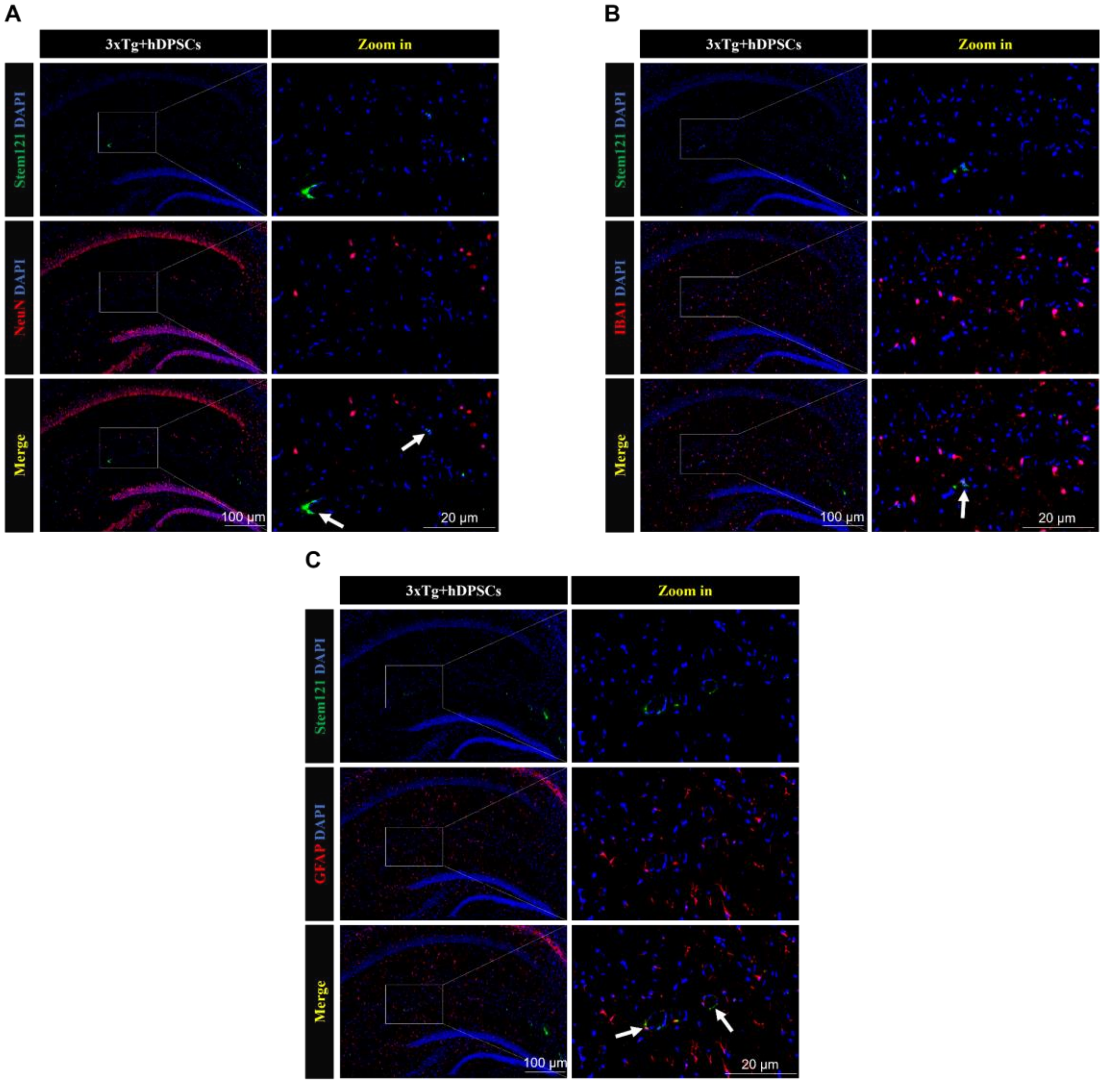

Figure S20. Specific human cell markers were detected in the hippocampus from mice 6 months after treatment. (A, B, C) Representative co-immunostaining of Stem121 (green) and NeuN (red)/IBA1 (red)/GFAP (red) to detect the differentiation status of hDPSCs in brain sections of the hippocampus from mice 6 months after therapy. Scale bar=100 μm (main images), 20 μm (magnified images, white arrows).

Table S1. Survey report of hDPSCs product by NIFDC

Inspection items	Standard stipulations	Inspection results
【physical test】		
Visible impurity	It should be a light yellow, semi-transparent suspension, non-breakable package, full label;	Meet the criteria
Volume (ml)	≥50	52
PH	6.5~7.8	6.9
Osmotic pressure (m0smol/kg)	250~310	279
【Cell number and viability】		
Total living cells (cells/bag)	2.0~3.0*10^7	2.9*10^7
Cell viability	≥90.0	97.5
【Cell identification】		
Cell strain identification (human STR map analysis)	It should be of single cell origin	AMEL:X; vWA:16<br>D21S11:31,32;<br>D18S51:13,17<br>PentaE:13,14;<br>D5S818:11,12;<br>D13S317:11; D7S820:12<br>D16S539:11; FGA:21,25<br>D3S1358:16; TH01:9<br>D8S1179:12,14; TPOX:8,11<br>CSF1PO:11,12; PentaD:9,11

Cell surfure antigen		
CD73 (%)	≥95.0	99.7
CD90 (%)	≥95.0	99.8
CD105 (%)	≥95.0	98.5
CD11b/CD19/CD34/CD45/HLA-DR (%)	≤2.0	0.5
【Sterility test】		
Membrane filtration method	Sterile growth	Meet the criteria
【Mycoplasma test】		
Culture method	Negative	Meet the criteria
Indicator cell culture method	Negative	Meet the criteria
【Bacterial endotoxin test】		
Kinetic chromogenic substrate	≤0.5	Meet the criteria

Abbreviation: NIFDC, National Institutes for food and drug control

Report No.SH202005736

Table S2. Primers and interference sequences used in this study

Gene	Forward primer	Reverse primer	Purpose
APP	5'-AGGACTGACCACTCGACCAG-3'	5'-CGGGGGTCTAGTTCTGCAT-3'	Genotyping
PS1	5'-AGGCAGGAAGATCACGTGTTCAAGTAC-3'	5'-CACACGCACACTCTGACATGCACAGGC-3'	Genotyping
Tau	5'-TGAACCAGGATGGCTGAG-3'	5'-TTGTCATCGCTTCCAGTCC-3'	Genotyping
IL-1β	5'-GCAACTGTTCCTGAACTCAACT-3'	5'-ATCTTTTGGGGTCCGTCAACT-3'	RT-PCR
TNF-α	5'-GACGTGGAACTGGCAGAAGAG-3'	5'-TTGGTGGTTTGTGAGTGTGAG-3'	RT-PCR
IL-6	5'-TAGTCCTTCCTACCCCAATTTCC-3'	5'-TTGGTCCTTAGCCACTCCTTC-3'	RT-PCR
IL-4	5'-GGTCTCAACCCCCAGCTAGT-3'	5'-GCCGATGATCTCTCTCAAGTGAT-3'	RT-PCR
IL-10	5'-GCTCTTACTGACTGGCATGAG-3'	5'-CGCAGCTCTAGGAGCATGTG-3'	RT-PCR
18s	5'-AACCCGTTGAACCCCATT -3'	5'-CCATCCAATCGGTAGTAGCG -3'	RT-PCR
IGF2	5'-GTGCTGCATCGCTGCTTAC-3'	5'-ACGTCCCTCTCGGACTTGG-3'	RT-PCR
NTF5	5'-TGAGCTGGCAGTATGCGAC-3'	5'-CAGCGCGTCTCGAAGAAGT-3'	RT-PCR
BDNF	5'-TCATACTTCGGTTGCATGAAGG-3'	5'-AGACCTCTCGAACCTGCCC-3'	RT-PCR
GDNF	5'-CCAGTGACTCCAATATGCCTG-3'	5'-CTCTGCGACCTTTCCCTCTG-3'	RT-PCR
Wnt8b	5'-CCCGTGTGCGTTCTTCTAGTC-3'	5'-AGTAGACCAGGTAAGCCTTTGG-3'	RT-PCR
DKK-1	5'-CTCATCAATTCCAACGCGATCA-3'	5'-GCCCTCATAGAGAACTCCCG-3'	RT-PCR
LRP6	5'-TTGTTGCTTTATGCAAACAGACG-3'	5'-GTTCGTTTAATGGCTTCTTCGC-3'	RT-PCR
Axin2	5'-TGACTCTCCTTCCAGATCCC-3'	5'-TGCCCACACTAGGCTGACA-3'	RT-PCR
Dvl2	5'-GGTGTAGGCGAGACGAAGG-3'	5'-GCTGCAAAACGCTCTTGAAATC-3'	RT-PCR
GSK3β	5'-TGGCAGCAAGGTAACCACAG-3'	5'-CGGTTCTTAAATCGCTTGTCCTG-3'	RT-PCR

β-catenin	5'-ATGGAGCCGGACAGAAAAGC-3'	5'-CTTGCCACTCAGGGAAGGA-3'	RT-PCR
TCF4	5'-CGAAAAGTTCCTCCGGGTTTG-3'	5'-CGTAGCCGGGCTGATTCAT-3'	RT-PCR
C-myc	5'-ATGCCCCTCAACGTGAACTTC-3'	5'-CGCAACATAGGATGGAGAGCA-3'	RT-PCR
Cyclin-D1	5'-GCGTACCCTGACACCAATCTC-3'	5'-CTCCTCTTCGCACTTCTGCTC-3'	RT-PCR

Table S3. Antibodies used in this study

Antibody	Cat No:	Company	Species	Application
p-Tau (AT8)	MN1020	Invitrogen	Mouse	WB/IF
p-Tau (Ser396)	44-752G	Invitrogen	Rabbit	WB/IF
Tau-5	Ab80579	Abcam	Mouse	WB
APP	25524-1-AP	Proteintech	Rabbit	WB
Aβ	Ab201060	Abcam	Rabbit	IF
Iba1	Ab178846	Abcam	Rabbit	IF
CD68	BA3638	Boster	Rabbit	IF
β-III Tubulin	Ab18207	Abcam	Rabbit	WB/IF
Nestin	MA1-110	Invitrogen	Mouse	WB/IF
NeuN	Ab177487	Abcam	Rabbit	WB/IF
GFAP	#80788S	CST	Rabbit	WB/IF
GAD67	MAB5406	Sigma	Mouse	IF
ChAT	AB144P	Sigma	Goat	IF
VGLUT1	135302	SYSY	Rabbit	IF
BDNF	28205-1-AP	Proteintech	Rabbit	WB
MAP2	67015-1-lg	Proteintech	Mouse	IF
Stem121	Y40410	Takara	Mouse	IF
Wnt8b	LS-C117181	Lifespan	Rabbit	WB
Axin2	20540-1-AP	Proteintech	Rabbit	WB

Dvl2	12037-1-AP	Proteintech	Rabbit	WB
Active β-catenin	#8814S	CST	Rabbit	WB
Total β-catenin	GB11015	Servicebio	Rabbit	WB
TCF4	13838-1-AP	Proteintech	Rabbit	WB
p-GSK3β(Ser9)	#9323S	CST	Rabbit	WB
GSK3β	Ab32391	Abcam	Rabbit	WB
C-myc	10828-1-AP	Proteintech	Rabbit	WB
Cyclin-D1	60186-1-Ig	Proteintech	Mouse	WB
BCL2	#3498S	CST	Rabbit	WB
Bax	50599-2-Ig	Proteintech	Rabbit	WB
Caspase 3	19677-1-AP	Proteintech	Rabbit	WB
Cleaved caspase-3	#9661S	CST	Rabbit	WB/IF
α-Tubulin	GB12200	Servicebio	Mouse	WB
GAPDH	GB11002	Servicebio	Rabbit	WB
